# Identification of transdiagnostic psychiatric disorder subtypes using unsupervised learning

**DOI:** 10.1038/s41386-021-01051-0

**Published:** 2021-06-14

**Authors:** Helena Pelin, Marcus Ising, Frederike Stein, Susanne Meinert, Tina Meller, Katharina Brosch, Nils R. Winter, Axel Krug, Ramona Leenings, Hannah Lemke, Igor Nenadić, Stefanie Heilmann-Heimbach, Andreas J. Forstner, Markus M. Nöthen, Nils Opel, Jonathan Repple, Julia Pfarr, Kai Ringwald, Simon Schmitt, Katharina Thiel, Lena Waltemate, Alexandra Winter, Fabian Streit, Stephanie Witt, Marcella Rietschel, Udo Dannlowski, Tilo Kircher, Tim Hahn, Bertram Müller-Myhsok, Till F. M. Andlauer

**Affiliations:** 1 Max Planck Institute of Psychiatry, Munich, Germany; 2 International Max Planck Research School for Translational Psychiatry, Munich, Germany; 3 Department of Psychiatry and Psychotherapy, Philipps-Universität Marburg, Marburg, Germany; 4 Center for Mind, Brain and Behavior (CMBB), Marburg, Germany; 5 Institute for Translational Psychiatry, Westfälische Wilhelms-Universität Münster, Münster, Germany; 6 Department of Psychiatry and Psychotherapy, University of Bonn, Bonn, Germany; 7 Institute of Human Genetics, University of Bonn School of Medicine & University Hospital Bonn, Bonn, Germany; 8 Institute of Neuroscience and Medicine (INM-1), Research Center Jülich, Jülich, Germany; 9 Centre for Human Genetics, University of Marburg, Marburg, Germany; 10 Central Institute of Mental Health, Medical Faculty Mannheim, Heidelberg University, Mannheim, Germany; 11 Munich Cluster for Systems Neurology (SyNergy), Munich, Germany; 12 Institute of Translational Medicine, University of Liverpool, Liverpool, UK; 13 Department of Neurology, Klinikum rechts der Isar, School of Medicine, Technical University of Munich, Munich, Germany; 14 Present Address: Global Computational Biology and Data Sciences, Boehringer Ingelheim Pharma GmbH & Co. KG, Biberach an der Riß, Germany

**Keywords:** Psychiatric disorders, Depression, Genetics, Translational research, Psychology

## Abstract

Psychiatric disorders show heterogeneous symptoms and trajectories, with current nosology not accurately reflecting their molecular etiology and the variability and symptomatic overlap within and between diagnostic classes. This heterogeneity impedes timely and targeted treatment. Our study aimed to identify psychiatric patient clusters that share clinical and genetic features and may profit from similar therapies. We used high-dimensional data clustering on deep clinical data to identify transdiagnostic groups in a discovery sample (*N* = 1250) of healthy controls and patients diagnosed with depression, bipolar disorder, schizophrenia, schizoaffective disorder, and other psychiatric disorders. We observed five diagnostically mixed clusters and ordered them based on severity. The least impaired cluster 0, containing most healthy controls, showed general well-being. Clusters 1–3 differed predominantly regarding levels of maltreatment, depression, daily functioning, and parental bonding. Cluster 4 contained most patients diagnosed with psychotic disorders and exhibited the highest severity in many dimensions, including medication load. Depressed patients were present in all clusters, indicating that we captured different disease stages or subtypes. We replicated all but the smallest cluster 1 in an independent sample (*N* = 622). Next, we analyzed genetic differences between clusters using polygenic scores (PGS) and the psychiatric family history. These genetic variables differed mainly between clusters 0 and 4 (prediction area under the receiver operating characteristic curve (AUC) = 81%; significant PGS: cross-disorder psychiatric risk, schizophrenia, and educational attainment). Our results confirm that psychiatric disorders consist of heterogeneous subtypes sharing molecular factors and symptoms. The identification of transdiagnostic clusters advances our understanding of the heterogeneity of psychiatric disorders and may support the development of personalized treatments.

## Introduction

Psychiatric disorders are typically diagnosed based on cross-sectional and longitudinal symptom profiles. However, different symptom patterns can result in the same diagnosis, and symptom arrays of different diagnoses may overlap, leading to heterogeneous clinical manifestations and trajectories. The risk for psychiatric disorders is multifactorial and influenced by the genetic background, early adverse experiences, and personality factors. Accounting for these risk factors may improve diagnostic accuracy. Common genetic variants confer an important share of psychiatric disorder risk, which can be quantified using polygenic scores (PGSs) [1]. Proportionally to the genetic risk load, a gradient of symptom severity may exist between healthy individuals and clinically diagnosed patients [2–5].

The wealth of available data and advances in machine learning intensified efforts to redefine disorder categories using data-driven methods. Previous studies stratified psychiatric disorders mostly by clustering single domains (e.g., psychometry [6–10], neuroimaging [11–16], biochemical markers [17], or genetics [18, 19]) or by analyzing patients from a single diagnosis (e.g., major depressive disorder (MDD) [5, 7, 11, 18–22] or schizophrenia (SCZ) [23–28]). Previous transdiagnostic clustering studies support the existence of diagnostically mixed subtypes across two [29–31] or more disorders [32–35]. However, these studies were limited by small samples and analyzed few disorders or variables [36, 37]. To our knowledge, Dwyer et al. [32] constitutes the largest published clustering study. It focused on psychosis, not covering the complete spectrum from healthy controls over affective to psychotic disorders. To assess the continuum between well-being and disease, clustering analyses profit from the inclusion of healthy controls, largely omitted in previous studies [7, 21, 29, 34].

In the present study, we applied a data-driven clustering approach to a large transdiagnostic patient/control sample. It encompassed healthy controls and patients diagnosed with MDD, bipolar disorder (BD), schizophrenia, schizoaffective disorder (SZA), or other psychiatric disorders (see below). Our study had the following two main aims: first, to use high-dimensional data clustering (HDDC) [38] to identify stable transdiagnostic clusters. Here, we used deep phenotypic data including psychopathology measures, personality traits, cognitive functioning, social functioning, attachment style, environmental exposures in childhood and youth, parental factors, and quality of life measures. Second, to characterize differences of clinical and genetic variables between clusters using supervised machine learning. Moreover, we analyzed the information gain of PGS compared with the family history of psychiatric disorders and replicated our clustering solution in an independent sample.

## Materials and methods

### Sample description

FOR2107 is an ongoing multi-center study recruiting patients via in- and outpatient services in Marburg and Münster, Germany; healthy subjects were recruited via newspaper advertisements [39]. Inclusion criteria for the cohort were comprehensive to ensure the recruitment of patients across different diagnoses, approximately representative for referrals to Western European psychiatric hospitals. The study protocols were approved by the ethics committees of the Medical Schools of the Universities of Marburg and Münster, following the Declaration of Helsinki, and all participants provided written informed consent. All subjects underwent a structured clinical interview for Diagnostic and Statistical Manual (DSM)-IV axis I disorders [40], administered by trained clinical raters.

All individuals recruited in the first phase of the study, i.e., whose data were available when we began the analyses, were eligible for the discovery sample (*N* = 1623), *N* = 855 independent individuals recruited subsequently were included for the replication. First, participants who had withdrawn their consent, with missing diagnosis, and relatives were excluded. Second, individuals with missing information in any of the variables used for clustering were excluded (Methods S1). Final sample sizes were *N* = 1250 (discovery) and *N* = 622 (replication). Age and diagnosis distributions differed between both samples (*p* = 0.01, *p* = 0.002, respectively), sex did not (*p* = 0.16). Among diagnostic groups, the proportions of healthy controls (*p* = 0.005) and MDD patients (*p* = 0.003) differed significantly (Tables 1 and S1).

**Table 1:** Characterization of the discovery sample and clusters.

Variable	Discovery	Cluster 0	Cluster 1	Cluster 2	Cluster 3	Cluster 4
*N*	1250	535	38	266	215	196
*Demographics*						
Age, mean (SD)	35.1 (13.0)	31.7 (11.9)	38.6 (13.9)	35.3 (12.7)	37.9 (13.7)	40.3 (12.8)
Male gender, *N* (%)	483 (39%)	205 (38%)	9 (24%)	106 (40%)	74 (34%)	89 (45%)
Years of education, mean (SD)	13.5 (2.6)	14.4 (2.4)	13.3 (2.6)	13.8 (2.6)	13.1 (2.8)	12.1 (2.7)
Living with partner, *N* (%)	277 (28%)	108 (20%)	7 (18%)	51 (19%)	62 (29%)	49 (25%)
BMI, mean (SD)	25.3 (5.5)	23.7 (4.3)	24.9 (5.1)	24.8 (4.9)	27.0 (6.6)	28.1 (6.4)
Family history (any psychiatric disorder), *N* (%)	533 (43%)	141 (26%)	16 (42%)	148 (56%)	105 (49%)	123 (63%)
*Diagnosis*						
Age at onset (AAO)*, mean (SD)	25.2 (11.9)	24.5 (9.9)	29.6 (13.3)	23.3 (11.5)	27.8 (12.8)	24.4 (11.4)
HC, *N* (%)	590 (47%)	448 (84%)	19 (50%)	78 (29%)	34 (16%)	11 (6%)
BD, *N* (%)	75 (6%)	9 (2%)	1 (3%)	15 (6%)	26 (12%)	24 (12%)
MDD, *N* (%)	477 (38%)	56 (10%)	17 (45%)	152 (57%)	147(68%)	105 (54%)
SCZ, *N* (%)	53 (4%)	4 (1%)	1 (3%)	8 (3%)	3 (1%)	37 (19%)
SZA, *N* (%)	25 (2%)	2 (0.3%)	0 (0%)	5 (2%)	2 (1%)	16 (8%)
Other, *N* (%)	30 (2%)	16 (3%)	0 (0%)	8 (3%)	3 (1%)	3 (2%)
*Quality of life (SF36), mean (SD)*						
General health	66 (23.4)	81.2 (14.0)	57.9 (26.6)	64.7 (19.8)	49.4 (20.7)	45.8 (21.3)
Mental health	64.3 (22.6)	81.4 (9.8)	55.6 (23)	59.4 (18.9)	46.4 (19.3)	45.6 (21.3)
*Depression and anxiety, mean (SD)*						
HAMA sum	7.3 (7.9)	2.2 (2.5)	9.7 (8.6)	7.6 (6.4)	13.1 (8.6)	14 (8.8)
HAMD sum	5.4 (6.6)	1.1 (1.6)	6.3 (6.7)	5.9 (5.7)	9.9 (7.0)	11 (7.5)
BDI sum	10.7 (10.8)	3.2 (3.3)	15.1 (12.8)	12.7 (9.6)	17.6 (10.1)	20.3 (11.7)
*Positive, negative, and manic symptoms, mean (SD)*						
SANS	5.7 (9.9)	0.6 (1.7)	5.2 (8.6)	6.9 (9.6)	8.0 (9.1)	15.6 (14.4)
SAPS	1.4 (5.2)	0.1 (0.6)	0.1 (0.4)	0.6 (1.6)	0.7 (1.8)	6.7 (11.5)
YMRS	1.2 (2.5)	0.5 (1.1)	0.8 (1.4)	1.1 (1.8)	1.3 (2)	2.9 (4.9)
*Maltreatment in childhood and youth (CTQ), mean (SD)*						
Emotional abuse	9.1 (4.7)	6.3 (1.7)	9.9 (4.9)	11.4 (4.2)	8.0 (3.0)	14.6 (5.9)
Emotional neglect	10.7 (5.2)	7.5 (2.6)	11.8 (5.4)	14.1 (4.1)	9.2 (3.5)	16.5 (5.4)
Physical abuse	6.2 (2.6)	5.3 (0.7)	6.4 (2.2)	6.4 (2)	5.5 (1.1)	9.5 (4.6)
Physical neglect	7.2 (2.7)	5.8 (1.4)	7.2 (2.2)	8.0 (2.2)	6.4 (1.6)	10.4 (3.7)
Sexual abuse	5.8 (2.5)	5.1 (0.4)	5.8 (2)	5.8 (1.9)	5.6 (1.7)	8.0 (4.9)

*BMI* body mass index, *AAO* age at onset of any psychiatric disorder (defined according to OPCRIT item 4) *not available for healthy controls, *HC* healthy controls, *BD* bipolar disorder, *MDD* major depressive disorder, *SCZ* schizophrenia, *SZA* schizoaffective disorder, *Other* other psychiatric diagnoses including anxiety, adjustment, and substance use disorders, *HAMA* Hamilton Anxiety Rating Scale, *HAMD* Hamilton Depression Rating Scale, *BDI* Beck Depression Inventory (BDI-II self-reported) *SANS/SAPS* Scale for the Assessment of Negative/Positive Symptoms, *SF36* 36-Item Short Form Survey.

Variable ranges and interpretations: quality of life [0–100, 0=disability]; HAMD 21 item [0–66, 0=not present]; HAMA [0–56, 0=not present]; BDI [0–63, 0=not present]; SAPS/SANS [0–86/0–80, 0=not present]; CTQ [5–25, 5=less severe experiences].

### Variables used for clustering and cluster description

**Fig. 1:**
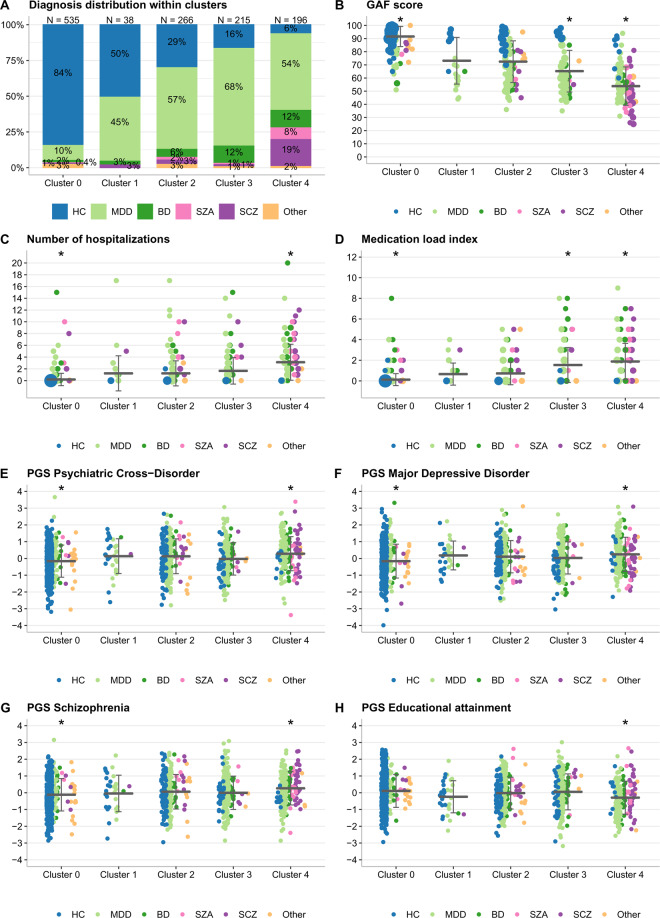
Cluster characterization in the discovery sample with clinical and genetic variables not used in the clustering pipeline. None of the variables shown in this Fig. 1 or Table S3 were included in the clustering pipeline. **B**–**H** a horizontal line represents the mean and the error bars indicate the standard deviation. The dot size is proportional to the number of individuals with the given value. Variables that were significant in the one-vs-all comparisons are marked with an asterisk sign. **E**–**H** show all PGS significant after Bonferroni correction (adjusted *p* < 0.05), tested using the Westfall and Young procedure (Methods S6), in either one-vs-all or one-vs-one analyses (Tables S12–S13). PGS were standardized by *Z* score transformation, the *y* axis unit is standard deviations. **A** The distribution of diagnoses within clusters. **B** The Global Assessment of Functioning (GAF) score, used for sorting clusters. Lower scores imply more severe impairment. **C** The number of times an individual was hospitalized. **D** The medication load index [59], reflecting the dose and variety of different medications taken. **E** Psychiatric cross-disorder PGS, significantly different in two one-vs-all analyses (lower in cluster 0, Bonferroni-corrected *p* = 0.004; higher in cluster 4, corrected *p* = 0.01). **F** MDD PGS, significantly different in two one-vs-all analyses (lower in cluster 0, *p* = 0.008; higher in cluster 4, corrected *p* = 0.04). **G** Schizophrenia PGS, significantly different in two one-vs-all analyses (lower in cluster 0, corrected *p* = 0.04; higher in cluster 4, corrected *p* = 0.01). **H** Educational attainment PGS, significantly different in one one-vs-all analysis (lower in cluster 4, corrected *p* = 0.004).

Fifty-seven baseline variables were used for clustering and the description of clusters (Fig. S1, Table S2). These variables were not directly used for establishing the diagnoses. Following a suggestion by Maj [36], we combined the assessment of symptoms and disease development at the current stage with variables capturing antecedent events, such as parental factors and early environmental factors, and concomitant variables such as cognitive functioning, social functioning (resilience), and personality traits. Several variables that were confounded with diagnostic groups, strongly differentiated psychiatric patients from healthy controls, or may have over-represented specific diagnostic aspects were excluded from clustering and retained for the post hoc characterization of clusters (Fig. 1A–D; for details, see Tables S2–S3). The self-reported family history of either any psychiatric disorder or specifically for MDD, BD, and SZA/SCZ was assessed for first-degree relatives and used for the genetic cluster characterization. We contrasted known with no/unknown family history.

### Genotyping and calculation of PGSs

Genotyping was conducted using the PsychArray BeadChip, followed by quality control and imputation, as described previously [41, 42] (Methods S2). Imputed genetic data were available for *n* = 1146 discovery-stage and *n* = 556 replication-stage individuals (Fig. S2). PGSs were calculated for ten disorders and traits using PRS-CS [43] (Methods S3) with training data from sufficiently powered, published genome-wide association studies: attention-deficit/hyperactivity disorder (ADHD) [44], autism spectrum disorder (ASD) [45], BD [46], psychiatric cross-disorder (CD) [47], educational attainment (EA) [48], extraversion [49], hedonic well-being [50], MDD [51], neuroticism [52], and schizophrenia [53].

### Clustering analysis

The clustering of discovery-stage scaled clinical variables was conducted by HDDC [38] using the *R* (v3.6.0) package *HDclassif* [54]. This package implements a subspace clustering algorithm based on the Gaussian mixture model framework, which allowed us to fit 14 different model types, corresponding to different regularizations for the cluster solutions. The clustering pipeline had four steps: finding the best fitting model type, finding the optimal cluster number, getting the final cluster solution, and assessing the solution’s stability (Methods S4 and Fig. S3). For the code used in this study, see https://github.com/hpelin/HDDC_transdiagnostic_clustering/.

### Characterization of clusters

In primary analyses, we characterized the clusters with the one-vs-all strategy [55], with one-vs-one pairwise comparisons in secondary analyses. Genetic analyses used 24 variables: 10 PGS, 4 family history, eight ancestry components, age, and gender. Merged with family history, the genetic sample size was *n* = 1137 (discovery) and *n* = 542 (replication).

We analyzed by supervised high-dimensional discriminant analysis (HDDA) [54, 56], which of the 57 variables used for clustering were most important for the cluster characterization (Methods S5).

Lasso-regularized regression [57] was used to predict cluster labels with genetic variables (Methods S5). Statistical testing was performed using the Westfall and Young method [58], controlling the family-wise error rate while accounting for the possible dependence structure of the analyzed variables. The obtained *p* values were subsequently corrected for the number of comparisons using Bonferroni’s method. For thus adjusted *p* values, a significance threshold *α* = 0.05 was used (Methods S6). We used multinomial regression to compare PGS with family history when predicting clusters (Methods S7).

### Replication analysis

We clustered the replication sample using the discovery-stage model parameters (Methods S8). Discovery-stage one-vs-all HDDA classification models were fit to the replication-stage clusters. Replication clusters were identified using the best discovery-stage model (balanced accuracy >70%).

After matching discovery and replication clusters, the discovery-stage genetic lasso models were projected to the replication sample.

## Results

### Model-based clustering analysis

The discovery-stage data set contained *N* = 1250 individuals with a mean age of 35.1 (SD = 13.0) years. For the distribution of diagnoses, see Table 1. Site-specific differences are reported in Table S4. We performed model-based HDDC using 57 baseline variables (Table S2). Our clustering pipeline (Fig. S4) identified five clusters (Fig. 1A), which were ordered by their average Global Assessment of Functioning (GAF) scores, from lowest (cluster 0) to highest severity (cluster 4) (Fig. 1B).

### Phenotypic characterization of clusters

Cluster 0 contained mostly healthy controls, whereas the other clusters were diagnostically more mixed (Fig. 1A). All clusters showed distinct profiles of diagnoses, symptoms, and environmental risk factors (Table 1 and S5).

**Fig. 2:**
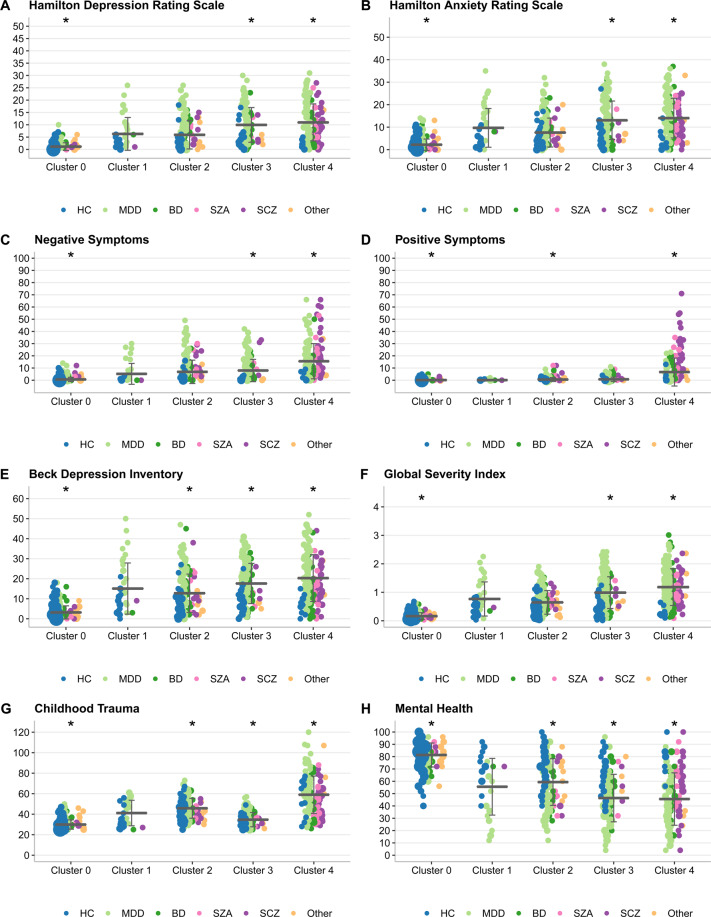
Cluster characterization in the discovery sample with variables used in the clustering pipeline. A horizontal line represents the mean, and the error bars indicate the standard deviation, whereas the dot size is proportional to the number of individuals with the given value. Variables that were significant in the one-vs-all comparisons are marked with an asterisk sign. **A** Hamilton Depression Rating Scale (HAMD, 21 items, clinician-administered), range 0–66, scores >7 indicate (mild) depression. **B** Hamilton Anxiety Rating Scale (HAMA), range 0–56, scores >17 indicate mild to moderate anxiety severity. **C** Scale for the Assessment of Negative Symptoms (SANS, sum score), range 0–80, a higher score indicates more severe negative symptoms. For subscales, see Table S3. **D** Scale for the Assessment of Positive Symptoms (SAPS, sum score), range 0–86, a higher score indicates more severe positive symptoms. For subscales, see Table S3. **E** Beck Depression Inventory (BDI-II, self-reported), range 0–63, scores >9 indicate (mild) depression. **F** Symptom Checklist–Global Severity Index, an index of overall psychological distress, range 0–4, higher scores reflect higher levels of psychopathological distress as well as a greater severity of self-reported symptoms. **G** Childhood Trauma Questionnaire sum score, range 25–125, a higher score indicates more experiences of childhood trauma. **H** SF36–Quality of life measurements–Mental health, range 0–100, high scores define a more favorable health state.

Individuals in cluster 0 (*n* = 535, 84% healthy controls) showed the overall best health and quality of life and exhibited the lowest severity in most symptom and risk scores (Figs. 1–2 and S5, Tables S3, S6, S7). The smallest cluster 1 (*n* = 38) included the highest rates of females (62%) and symptomatic controls without a diagnosis (50%), who reported reduced general and mental health and increased anxiety and depression symptoms (Table 1, Fig. 2). Individuals in cluster 2 showed average general health scores but reduced mental health and parental bonding and elevated emotional maltreatment scores (Tables 1 and S7, Figs. 2 and S5). Cluster 3 had the highest rate of affective diagnoses with high depression and anxiety levels (Fig. 1A, Table 1); its members reported substantially reduced general and mental health. The mean childhood maltreatment scores in cluster 3 were lower than in clusters 1, 2, and 4 (Table 1). Cluster 4 (*n* = 196) featured most patients diagnosed with SZA and schizophrenia (Fig. 1A). Individuals in cluster 4 were characterized by the highest severity in many dimensions used for clustering (Tables 1 and S7, Fig. 2) and in additional variables examined post hoc, such as hospitalization and medication load index [59] (Fig. 1, Table S3).

As a secondary analysis, we characterized MDD patients within the five clusters to assess the heterogeneity of this large diagnostic group and identified distinct phenotypic signatures of MDD patients in each cluster (Tables S8–S9).

### Genetic characterization: variable selection

We conducted lasso regularized regression to predict cluster assignments using genetic variables, i.e., ten PGS and four self-reported family history assessments. Prediction performances were highest for the two extreme clusters 0 and 4 (cluster 0 vs. 4: area under the receiver operating characteristic curve (AUC) = 81%, sensitivity = 75%, specificity = 75%; cluster 0 vs. all: AUC = 71%, sensitivity = 66%, specificity = 66%; cluster 4 vs. all: AUC = 73%, sensitivity = 67%, specificity = 67%, Table S10). Lasso selected seven variables when comparing cluster 0 against all others and 16 for cluster 4 (Table 2). In both cases, the self-reported family history achieved larger effect sizes than PGSs of psychiatric disorders. For lasso summary statistics, see Table S11.

**Table 2:** Genetic characterization of the discovery clusters.

Category	Lasso model coefficients	Cluster 0 vs. all	Cluster 1 vs. all	Cluster 2 vs. all	Cluster 3 vs. all	Cluster 4 vs. all
Demographic	Age	−0.335	0.077	0	0.181	0.381
	Gender	0	−0.188	0	−0.081	0.139
Ancestry components	AC1	0	0	0	0.081	0
	AC2	0	0	0	0.013	0
	AC3	0	0.061	0	0.003	−0.063
	AC4	0	0.122	0	0	−0.144
	AC5	0	0.086	0	−0.03	0.083
	AC6	0	0	0	0	−0.004
	AC7	0	0	0	−0.069	0
	AC8	0	0	0	−0.003	0
Family history	Any psychiatric disorder	−0.445	0	0.159	0	0.338
	BD	−0.007	−0.06	0	0	0.238
	MDD	0	0	0.044	0.095	0
	Schizophrenia	0	0	0	0.029	0.051
Polygenic scores	Cross psychiatric disorder	−0.093	0.102	0	−0.04	0.093
	ADHD	0	0	0	−0.018	0
	ASD	0	0	0	0	0
	BD	0	−0.199	0	0.094	0
	MDD	−0.074	0.05	0	0	0.112
	Schizophrenia	0	0	0	0	0.167
	Educational attainment	0.043	−0.094	0	0.002	−0.269
	Extraversion	0	0	0	0.023	0.107
	Hedonic well-being	0	0	0	−0.018	−0.128
	Neuroticism	−0.009	0	0.05	0.095	−0.016

The table shows coefficients from lasso regularized regression models, fit to the full discovery sample, as explained in the Methods S5. A positive sign indicates that the given predictor variable is more likely to be higher in the respective cluster.

For prediction performances and summary statistics, please see Tables S10 and S11, respectively. For further genetic analyses, please see Tables S12–S15.

*AC* ancestry components, *BD* bipolar disorder, *MDD* major depressive disorder, *ADHD* attention-deficit/hyperactivity disorder, *ASD* autism spectrum disorder.

### Genetic characterization: statistical significance

We used Westfall and Young’s method to assess the significance of genetic variables. One-vs-all comparisons of clusters 0, 2, and 4 identified the following significant genetic variables (Table S12 and Fig. 1E–H): Cluster 0 was characterized by a lower family history of MDD, BD, and any psychiatric disorder (each adjusted *p* = 0.004) and lower cross-disorder (*p* = 0.004), MDD (*p* = 0.008), and schizophrenia (*p* = 0.04) PGS. Cluster 2 was characterized by a higher family history of any psychiatric disorder (*p* = 0.005) and MDD (*p* = 0.03). Cluster 4 showed a higher family history of any psychiatric disorder (*p* = 0.004) and higher cross-disorder (*p* = 0.01), schizophrenia (*p* = 0.01), and MDD (*p* = 0.04) PGS, as well as lower PGS for educational attainment (*p* = 0.004). Pairwise comparisons resulted in significant differences between four cluster pairs (Table S13). Cluster 4 MDD patients showed significantly higher ADHD (*p* = 0.01) and lower educational attainment PGS (*p* = 0.005) than MDD patients from the other clusters (Table S8 and Fig. S6A, B). As a sensitivity analysis, we compared PGS between diagnostic labels (Table S14).

### Genetic characterization: assessment of the information gain

The inclusion of PGSs and ACs in a multinomial cluster prediction model yielded an increase of *R*^*2*^ = 11.7% over a null model without genetic variables (Table S15). The family history alone improved the *R*^*2*^ by 10.8% over the null model; a model with both family history and ACs showed a gain of *R*^*2*^ = 13.9%. PGSs, ACs, and family history together increased *R*^*2*^ by 20.3%. PGSs improved the model containing family history and ACs significantly (likelihood ratio test *p* = 5 ⨯ 10^−5^).

### Replication of the clustering analysis

**Fig. 3:**
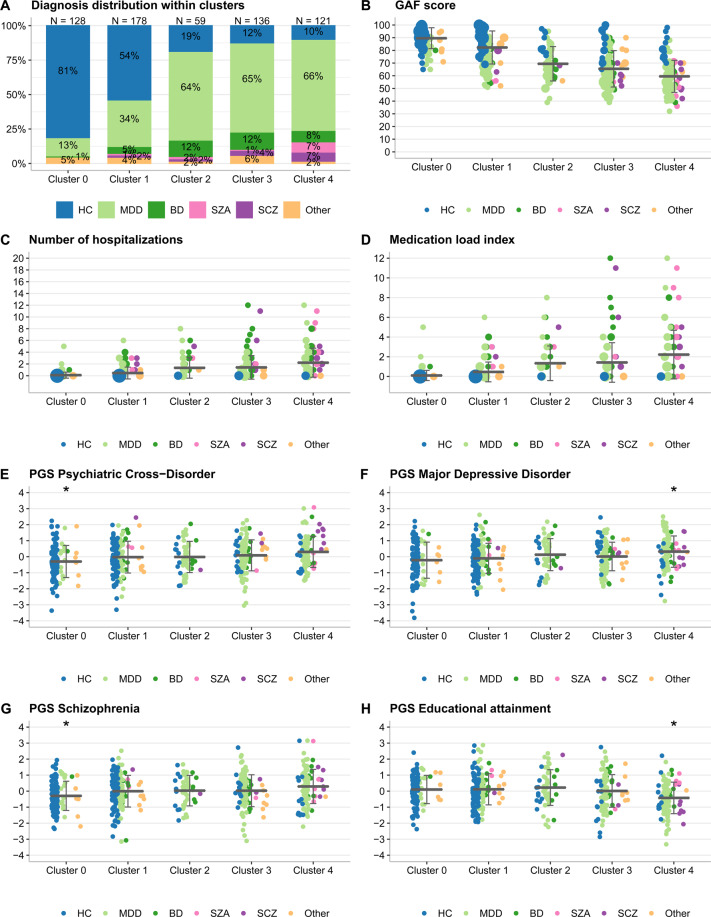
Cluster characterization in the replication sample with clinical and genetic variables not used in the clustering pipeline. **B**–**H** a horizontal line represents the mean, and the error bars indicate the standard deviation, whereas the dot size is proportional to the number of individuals with the given value. **E**–**H** show all PGS that were significant after Bonferroni correction (adjusted *p* < 0.05) in either one-vs-all or one-vs-one analyses using the Westfall and Young procedure (Methods S6) in the discovery-stage analysis. All *p* values for the full replication sample are shown in Tables S19 and S20. PGS were standardized by *Z* score transformation, the *y* axis unit are standard deviations. **A** The distribution of diagnoses within clusters. **B** The Global Assessment of Functioning (GAF) score, used for sorting clusters. Lower scores imply more severe impairment. **C** The number of times an individual was hospitalized. **D** Medication load index [59], reflecting dose and variety of different medications taken. **E** Psychiatric cross-disorder PGS, replicated for the comparison cluster 0-vs-all (corrected *p* = 0.03). **F** Major depressive disorder PGS, replicated for the comparison cluster 4-vs-all (corrected *p* = 0.01). **G** Schizophrenia PGS, replicated for the comparison cluster 0-vs-all (*p* = 0.005). **H** Educational attainment PGS, replicated for the comparison cluster 4-vs-all (corrected *p* = 0.005).

The replication data set contained *N* = 622 individuals with a mean age of 36.3 (SD = 12.6) years (Table S1). HDDA models matched all but the smallest cluster 1 between discovery and replication samples (Fig. S7). The matched replication clusters followed the same severity ranking as the discovery-stage clusters, and many variables showed highly similar severity patterns (Fig. 3, Tables S1, S16–S17).

The discovery-stage genetic lasso regression models applied to the replication clusters showed an AUC = 63%, sensitivity=60%, specificity=60% for cluster 0 vs. all and an AUC = 68%, sensitivity=67%, specificity=66% for cluster 4 *vs*. all, similar to the discovery sample. Further projections of five pairwise models yielded AUCs >60% (Table S18). As observed in the discovery sample, cross-disorder (adjusted *p* = 0.03) and schizophrenia (*p* = 0.005) PGS were significantly lower in the replication-stage cluster 0 (Table S19 and Fig. 3E, G). For cluster 4, the MDD PGS (*p* = 0.01) was higher and the educational attainment PGS lower (*p* = 0.005), confirming the discovery-stage results (Fig. 3F, H). Also schizophrenia and cross-disorder PGS were, as in the discovery stage, higher in cluster 4, but these associations showed only nominal significance and did not pass correction for multiple testing. In pairwise comparisons, replicated PGS associations included the associations of schizophrenia, cross-disorder, and educational attainment PGS when comparing cluster 0 with 4 (Table S20). MDD individuals in cluster 4 had, as in the discovery stage, significantly lower EA PGS than MDD patients in other clusters, whereas the association of ADHD PGS for MDD patients in cluster 4 did not replicate (Fig. S6C–D).

## Discussion

The symptoms and disease courses of patients diagnosed with any given major psychiatric disorder are highly heterogenous, suggesting ethiopathological differences between patients sharing the same diagnosis. The classification and treatment of psychiatric disorders rely on a nosological approach that does not necessarily reflect the disorders’ molecular etiology.

In the present study, we characterized subgroups in a large transdiagnostic cohort, including healthy controls, after clustering 57 multi-modal phenotypic variables. By combining model-based clustering with supervised machine learning for cluster characterization, we generated robust and replicable outcomes. Furthermore, we described clusters using genetic variables.

### Comparison of clusters to a severity continuum

We identified five diagnostically mixed clusters, which were ranked along a continuous severity scale. Cluster 0 contained mostly healthy controls and was distinguished by the lowest severity in many measures—from the lowest maltreatment factors, depression level, and positive symptoms to the highest quality of life scores. Cluster 4 had the highest share of schizophrenia and SZA patients and showed the highest severity in many variables not used for the clustering, e.g., the medication load index [59] and the number of hospitalizations. Clusters 1–3 ranged between these two extremes and differed mostly in different levels of maltreatment, depression and antidepressant use, daily functioning, and parental bonding.

Using principal component analysis and *SigClust* [60], we could not find support for the hypothesis that a simple severity component explains our clustering best (Results S1, Table S21). The five identified categorical clusters thus rank along but do not exactly correspond to a severity continuum.

Importantly, all but the smallest of these clusters were replicated in an independent sample. Given that the proportions of diagnoses in the replication sample differed, the replication of these clusters and their characteristics, especially the severity spectrum and genetic variables, is remarkable. It underlines the stability of the cluster solution and indicates that our approach did not suffer from overfitting in the discovery sample.

### Characterization of potential disorder subtypes

Compared with DSM-IV diagnostic categories, our cluster solution surpassed diagnostic boundaries mostly for MDD and BD, while patients diagnosed with schizophrenia and SZA were primarily grouped in the high-severity cluster 4. This finding confirms etiological similarities between the affective disorders MDD and BD, distinguishing them from predominantly psychotic disorders [61, 62]. Inclusion of more schizophrenia patients may have led to better discrimination of schizophrenia subtypes, as identified in previous studies [32, 63].

MDD patients were present in all five clusters, suggesting that different disorder subtypes or stages were captured. Interestingly, 80% of MDD patients in the lowest severity cluster 0 were in remission of either single or recurrent MDD at the assessment time (coded according to the DSM). Hence, their present clinical presentation was similar to healthy individuals. MDD patients in cluster 1 might represent a reactive depression subtype, with similarities to burnout (i.e., a high somatization level and life stress, low energy, and a higher age of disorder onset). MDD cases in cluster 2, with the lowest average age of onset, might suffer from exogenous depression triggered by external stressors (maltreatment and neglect in childhood). Interestingly, this cluster also contained the highest ratio of BD type-II/type-I patients (Table S22). However, these patients also showed a high genetic predisposition for depression, with 48% reporting an MDD family history. In cluster 3, MDD patients showed a low influence of adverse environmental factors and high parental bonding, similar to cluster 0. Nevertheless, their quality of life was impacted negatively by illness—cluster 3 MDD patients showed low energy and experienced limitations in role activities because of physical and emotional health problems.

Consistent with the strong presence of schizophrenia patients, cluster 4 MDD patients exhibited depression with psychotic features, showing higher positive symptoms and more antipsychotic intake. These MDD patients had significantly higher ADHD PGS than MDD patients in other clusters (*p* = 0.009). Previous studies have identified correlations between ADHD in childhood and the development of other severe psychiatric disorders, especially schizophrenia, in adulthood [64–66]. Although not available at present, a retrospective assessment of ADHD symptoms during childhood in cluster 4 MDD cases might shed further light on this correlation. MDD (and BD) patients in cluster 4 showed significantly more psychotic features than MDD/BD cases in other clusters (Table S23).

### Characterization of healthy controls

Healthy controls distributed across clusters 1–4 showed isolated symptoms similar to the psychiatric patients in these clusters (Table S24). The number of healthy controls decreased with cluster severity. Apparently, the symptoms of these healthy individuals were not sufficiently severe to generate a clinically relevant presentation of any psychiatric disorder fitting the currently used nosology. For example, these individuals may have only experienced short-term symptoms, e.g., resulting from a recent adverse life event. Indeed, healthy controls in cluster 4 showed a negative events score of 21, higher than the median of any other disorder group in the clusters showing high impairment. Alternatively, they might develop a disorder later in life; with a mean age of 32, the healthy individuals were younger than the average assessed patients.

Analyses of genetic differences between healthy controls assigned to different clusters identified nominally significant differences for the ADHD PGS, similarly to the MDD subtype analysis (Table S24 and Fig. S8). Follow-up assessments of the longitudinal FOR2107 study may reveal whether a higher share of healthy controls mapping to the more severe clusters will develop a disorder over time.

### Moving beyond classical diagnostic groups

Possibly, the current diagnostic criteria do not capture the whole illness spectrum. Our study might thus contribute to improved diagnostic criteria, as envisioned by the Research Domain Criteria (RDoC) project [67]. In agreement with the RDoC concept, we included variables from different domains, including behavioral tests for evaluating cognitive functioning. Although cluster 4 patients showed the lowest cognitive functioning, these differences did not substantially contribute to the clustering, possibly due to the “reliability paradox” of behavioral tests [68, 69]. These tests are particularly sensitive to situational modulators like attention and motivation as well as experience and learning effects.

MDD and BD patients were distributed over all five clusters, with similar shares of individuals mapping to clusters 2–4. Although most healthy controls were assigned to cluster 0 and most schizophrenic patients to cluster 4, 24% of healthy controls were not in cluster 0, and 30% of schizophrenia patients not in cluster 4. Among MDD patients, 22% were assigned to the high-severity cluster 4. The spread of MDD patients across all clusters supports the hypothesis that classical diagnostic groups may be inferior to a symptom-derived grouping of patients.

### Characterization of clusters using PGSs

Supervised analyses of genetic variables confirmed that PGS added information to cluster comparisons beyond what could be assessed using the family history of disorders. The slight increase of explained variance conveyed by ancestry information underlined the highly polygenic nature of psychiatric disorders. Interestingly, a recent study highlighted the benefits of adding both the family history and PGS to prediction models [70]. Psychiatric cross-disorder, schizophrenia, and MDD PGS were significantly higher in the most severe cluster 4 compared with cluster 0, whereas educational attainment PGS were lower—corresponding to effect directions reported in previous studies [47, 51, 53, 61, 71]. Although PGS are still far from routine clinical use in psychiatry, they might be used for patient stratification in the future [1, 5, 63, 72].

Interestingly, genetic PGS analyses on diagnostic categories produced different results from analyses of cluster labels. For example, cluster 0 showed higher educational attainment and lower neuroticism PGS, both of which did not differ significantly between healthy controls and the other probands. Similarly, cluster 4 showed an association with several PGS while schizophrenia patients only showed increased schizophrenia PGS. These genetic differences corroborated the transdiagnostic nature of the identified clusters.

### Comparison to previous clustering studies

To our knowledge, the present study is the first to cluster multidomain profiles of clinical variables across psychiatric disorders and including healthy controls. Nevertheless, the cluster profiles and identified severity spectrum partially aligns with previous findings. A transdiagnostic study identified a cluster containing mainly healthy controls and exhibiting the lowest symptom scores in the observed dimensions [34], likely corresponding to our cluster 0. Our highly impaired cluster 4, with its high percentage of schizophrenic patients, low functioning, and significantly lower EA PGS, may correspond to the severe psychosis subtype from a previous study [32]. Moreover, a single-disorder subtyping study [7] detected five clusters of MDD, with one subgroup showing an absence of many symptoms, similar to our cluster 0. Furthermore, our results highlight the correlation of various measures of childhood trauma, adverse experiences, and lack of support with illness severity, positive symptoms, hospitalizations, and the need for more intensive treatment. Several prior studies support such a correlation [30, 73–76].

### Limitations

Most psychiatric patients in our transdiagnostic study have been diagnosed with MDD, with only a smaller share of other, especially psychotic diagnoses. Such a distribution approximately resembles known differences in prevalence between mental health disorders in the general population. Although the high number of MDD patients allowed for a detailed description of depression subtypes, a similarly detailed characterization was not possible for psychotic disorders, which concentrated in cluster 4. Future transdiagnostic studies applying our clustering approach with more psychotic patients could focus on BD and schizophrenia subtypes, as suggested by previous single-disorder studies [25, 28].

Although we observed no overrepresentation of depression-related variables in our analysis (Results S2), we cannot entirely exclude that the variable selection influenced the obtained clustering solution. Furthermore, the diagnostic groups differed in demographic variables like age and sex, resulting in corresponding differences between clusters (Table 1, Results S2).

Moreover, although we used independent individuals for the replication data set, these probands were subsequently recruited within the same study as the discovery-stage sample. Accordingly, the proportions of healthy controls and MDD patients differed between the discovery and replication samples, limiting their comparability. We conducted the quality control of the phenotypic and genetic data jointly for both data sets, introducing minor dependencies. Furthermore, the replication sample was smaller than the discovery sample, attenuating its statistical power.

Finally, the clustering algorithm we used relied on discrete categorization and a given number of clusters. Assuming the existence of a symptom continuum from healthy to severe mental illness, future studies might consider applying methods incorporating the notion of a continuum into the global objective function [77].

## Conclusion

In conclusion, our study constitutes a data-driven, computational approach to psychiatric disorder stratification that surpasses existing diagnostic categories and integrates different domain profiles.

Our analyses support the hypothesis that psychiatric disorders consist of heterogeneous subtypes that share etiological factors and symptoms. We have demonstrated the importance of stratifying symptoms and disorder subtypes that can be ranked according to their severity. Individuals formally diagnosed with the same disorder differ in their specific impairment. Furthermore, their symptoms may partly overlap with symptoms exhibited by patients with different diagnoses, highlighting the need for symptom- instead of diagnosis-specific treatment. Our transdiagnostic clustering approach may advance the understanding of the heterogeneity within and between psychiatric disorders. If applied to further cohorts, it may help the identification of patient groups sharing clinical features and thus profiting from similar treatments. The identification of such groups can lead to the development of more appropriate diagnoses, targeted treatment options, and prediction models for the disease course. Future assessments in FOR2107 and other longitudinal studies can reveal whether patients mapping to the different clusters show similar disease courses and treatment responses.

## Funding and disclosure

The Forschungsgruppe/Research Unit FOR2107 study was funded by the German Research Foundation (DFG): grants KI 588/14-1, KI 588/14-2 to T.K.; DA 1151/5-1, DA 1151/5-2 to UD; NE 2254/1-2 to I.N.; HA 7070/2-2, HA 7070/3, HA 7070/4 to T.H.; MU1315/8-2 to B.M.M.; RI 908/11-1, RI 908/11-2 to M.R.; NO 246/10-1, NO 246/10-2 to M.M.N.; WI 3439/3-1, WI 3439/3-2 to S.W. The study was supported by the German Federal Ministry of Education and Research (BMBF), through the Integrated Network IntegraMent, under the auspices of the e:Med programme (grants 01ZX1314A, 01ZX1614A to M.M.N.; 01ZX1314G, 01ZX1614G to M.R.; 01ZX1614J to B.M.M.), through BMBF grants 01EE1406C to M.R. and 01EE1409C to M.R. and S.H.W., and through ERA-NET NEURON, “SynSchiz - Linking synaptic dysfunction to disease mechanisms in schizophrenia - a multilevel investigation“ (01EW1810 to M.R.) and BMBF grants 01EE1409C and 01EE1406C to M.R. and S.H.W. Till Andlauer was supported by the BMBF through the DIFUTURE consortium of the Medical Informatics Initiative Germany (grant 01ZZ1804A) and the European Union’s Horizon 2020 Research and Innovation Programme (grant MultipleMS, EU RIA 733161). The authors have nothing to disclose. Open Access funding enabled and organized by Projekt DEAL.

## Supplementary information

Supplementary Material

Supplementary Tables
