# Supplementary material for: Identification of transdiagnostic psychiatric disorder subtypes using unsupervised learning
Source: Neuropsychopharmacology. 2021 Jun 14;46(11):1895–905. doi: 10.1038/s41386-021-01051-0 (PMC8429672; doi:10.1038/s41386-021-01051-0)
Supplement: Supplementary file 1 — Supplementary Material [file 41386_2021_1051_MOESM1_ESM.pdf]

Identification of transdiagnostic psychiatric disorder subtypes using unsupervised learning – Supplemental Material

Methods S1. Individual-level quality control ... 2
Methods S2. Genotyping, quality control, and imputation ... 2
Methods S3. Calculation of polygenic scores ... 3
Methods S4. Clustering analysis ... 3
Methods S5. Characterization of clusters ... 4
Methods S6. Statistical testing ... 4
Methods S7. Assessment of the information gain by PGS ... 4
Methods S8. Replication analysis ... 5
Results S1. Follow-up analyses of the severity continuum ... 5
Results S2. Assessment of the variable selection on the clustering solution ... 5
Table S1. Characterization of the replication sample and clusters ... 6
Table S2. Variables used in the clustering procedure ... 6
Table S3. Characterization of the discovery sample with variables not used for clustering ... 6
Table S4. Site-specific differences across clusters and diagnoses ... 7
Table S5. Most important features from the HDDA analysis ... 8
Table S6. Lasso - clinical variables average coefficients ... 8
Table S7. Characterization of the discovery sample and its clusters regarding variables used in the clustering process ... 8
Table S8. MDD subtype analysis ... 9
Table S9. *One-vs-all* HDDA classification analysis using only MDD-diagnosed patients ... 9
Table S10. Metrics of genetic lasso regularized regression prediction models ... 10
Table S11. Genetic lasso regularized regression prediction models: summary statistics ... 10
Table S12. Significance testing of genetic analyses with the Westfall and Young procedure – *one-vs-all* comparisons ... 11
Table S13. Significance testing of genetic analyses with the Westfall and Young procedure – *one-vs-one* comparisons ... 12
Table S14. Genetic analysis with diagnostic (DSM-IV) labels ... 13
Table S15. Assessment of the PGS information gain (cluster labels) ... 15
Table S16. Significance testing with the Westfall and Young procedure – comparison of discovery-replication pairs ... 16
Table S17. Characterization of the replication sample and its clusters with variables used in the clustering process ... 16
Table S18. Metrics of genetic lasso regularized regression models in the replication sample ... 17
Table S19. Replication – Significance testing with the WY procedure, *one-vs-all* comparisons .. 17
Table S20. Replication – Significance testing with the WY procedure, *one-vs-one* comparisons 18
Table S21. PCA analysis with variables used for clustering ... 19
Table S22. Bipolar disorder subtypes across clusters. ... 20
Table S23. Assessing psychotic symptoms of MDD and BD patients in cluster 4 ... 20
Table S24. Healthy Controls – clinical assessment ... 20
Figure S1. Correlation plot of variables used for clustering ... 21

**Figure S2.**	Population substructure (genetic ancestry components)	22
**Figure S3.**	Overview of the clustering pipeline	24
**Figure S4.**	Results from the clustering pipeline	25
**Figure S5.**	Distributions of the most important variables per cluster	26
**Figure S6.**	Distribution of PGS for MDD patients	29
**Figure S7.**	Discovery-stage HDDA models projected to the replication sample	30
**Figure S8.**	Distribution of ADHD PGS for healthy controls	31

Full FOR2107 acknowledgements 32

Supplementary Materials References 33

Methods S1. Individual-level quality control

Discovery sample
Of 1623 individuals eligible for the discovery sample, three were excluded because they withdrew their consent and one for missing diagnostic information from all subsequent analysis.
Next, 47 pairs of genetic relatives were identified in PLINK using the command *--genome*. Of each pair showing a PI-HAT ≥12.5, the individual with the higher genotyping rate was kept in the analyses. Finally, 322 individuals with missing information in any of the 57 variables used for clustering were excluded. The final discovery sample consisted of 1250 individuals, including 590 healthy controls, 477 MDD, 75 BD, 25 SZA, and 53 SCZ patients, and 30 individuals with other diagnoses disorders including anxiety disorders, adjustment disorders, and substance use disorders.

Replication sample
Of 855 individuals eligible for the replication dataset, one was excluded because of a withdrawn consent and two for missing diagnostic information. Next, 27 relatives were removed using the same procedure as applied for the discovery sample. Finally, 201 individuals were excluded due to missing information in any of the variables used for clustering. The final replication sample for the analysis consisted of 622 individuals, including 240 healthy controls, 283 MDD, 44 BD, 13 SZA, and 17 SCZ patients, and 25 individuals with other diagnoses including anxiety, adjustment, and substance use disorders.

Methods S2. Genotyping, quality control, and imputation
Genotyping was conducted using the Infinium PsychArray BeadChip, as described previously [1]. The quality control (QC) of genetic data was conducted in PLINK v1.90b6.10 [2] and R v3.5.2, as described previously [3]. Pre-imputation QC of genotype data consisted of the following steps:

1. Removal of SNPs with call rates <98% or a minor allele frequency (MAF) <1%
2. Removal of individuals with genotyping rates <98%
3. Removal of sex mismatches
4. Removal of genetic duplicates
5. Removal of cryptic relatives with pi-hat≥12.5
6. Removal of genetic outliers with a distance from the mean of >4 SD in the first eight multidimensional scaling (MDS) ancestry components
7. Removal of individuals with a deviation of the autosomal or X-chromosomal heterozygosity from the mean >4 SD
8. Removal of non-autosomal variants
9. Removal of SNPs with call rates <98% or a MAF <1% or Hardy-Weinberg Equilibrium (HWE) test *p*-values $<1\times10^{-6}$
10. Removal of A/T and G/C SNPs
11. Update of variant IDs and positions to the IDs and positions in the 1000 Genomes Phase 3 reference panel
12. Alignment of alleles to the reference panel
13. Removal of duplicated variants and variants not present in the reference panel

For the calculation of ancestry components (used to determine genetic outliers and as covariates in the analyses), pre-imputation genotype data were used. Additional variant filtering steps were: removal of variants with a MAF <0.05 or HWE *p*-value $<10^{-3}$; removal of variants mapping to the extended MHC region (chromosome 6, 25-35 Mbp) or to a typical inversion site on chromosome 8 (7-13 Mbp); linkage disequilibrium (LD) pruning (command `--indep-pairwise 200 100 0.2`). Next, the pairwise identity-by-state (IBS) matrix of all individuals was calculated using the command `--genome` on the filtered genotype data. Multidimensional scaling (MDS) analysis was performed on the IBS matrix using the eigendecomposition-based algorithm in PLINK v1.90b6.10.

After imputation, variants with a MAF <1%, an HWE test $p<1\times10^{-6}$, and an INFO metric <0.8 were removed. Imputation was conducted using SHAPEIT v2 (r837) [4], IMPUTE2 v2.3.2 [5, 6], and the 1000 Genomes Phase 3 reference panel.
In total, imputed genetic data were available for 1,416 individuals in the discovery and 556 individuals in the replication sample. These individuals were imputed as part of a larger dataset with N=2,248. Variants before QC: 596,861; variants after QC: 284,691; variants after imputation: 8,565,143.

Methods S3. Calculation of polygenic scores

Ten PGSs were calculated for the following disorders and traits, using training summary statistics from published genome-wide association studies (GWASs): ADHD [7] with 19,099 cases and 34,194 controls, ASD [8] with 18,382 cases and 27,969 controls, BD [9] with 20,352 cases and 31,358 controls, psychiatric cross-disorder (CD) [10] with 162,151 cases and 276,846 controls, educational attainment (EA) [11] with 293,723 individuals, extraversion [12] with 63,030 individuals, hedonic well-being (HWB) [13] with 221,575 individuals, MDD [14] with 170,756 cases and 329,443 controls, neuroticism [15] with 329,821 individuals, and SCZ [16] with 40,675 cases and 64,643 controls. PGSs were calculated using the PRS-CS [17] method that employs Bayesian regression to infer PGS weights while modeling local linkage disequilibrium (LD) patterns using the 1000 Genomes EUR reference panel. All training GWAS variants with an INFO metric <0.6, a MAF <1%, or which were not present in the FOR2107 imputation were removed before estimating PRS-CS weights. The global shrinkage parameter was fixed to $\varphi=0.01$, appropriate for highly polygenic traits. PGSs were calculated in *R* using the PRS-CS weights and imputed dosage data, as described previously [18].

Methods S4. Clustering analysis

The clustering analysis was conducted in *R* v3.6.0 using the discovery sample. Clinical variables were scaled and submitted to the HDDC algorithm [19] implemented in the *R* package *HDclassif* [20]. This package implements a subspace clustering algorithm based on the Gaussian mixture model framework, which allowed us to fit 14 different models, corresponding to different regularizations for the cluster solutions, as explained in detail elsewhere [19]. In order to choose the model best fitting for our data, HDDC was run 100 times using cross-validation. In each run, 80% of the discovery dataset was randomly resampled without replacement. The best-fitting model, AkBkQkDk, was chosen using the Integrated Completed Likelihood (ICL) criterion [21] (Suppl. Fig. S4A).

The model AkBkQkDk has the following characteristics:

Ak: The classes have different parameters but there is only one parameter per class
Bk: Each class has its proper noise
Qk: All classes have their proper orientation matrix
Dk: The dimensions are free and proper to each class

In the context of the data, the optimal model that was chosen assumes that there is a cluster-specific variance (Ak), cluster-specific noise (Bk), and that clusters can have different orientations (Qk). Since the HDDC algorithm is the subspace clustering algorithm based on the assumption that the clusters live in the subspaces of a lower dimension than the initial one, Dk is in this context considered to be the dimension of the cluster-specific subspace. For more theoretical details, see Bouveyron *et al.* [19].

After the choice of the optimal model (AkBkQkDk), the optimal number of clusters was determined using a leave-one-out jackknife resampling procedure, considered to well-suited for depicting the overall variation of the relevant metrics [22]. Here, the potential clustering solutions from K=2 to K=15 clusters were tested (Suppl. Fig. S4B). In this procedure, the model was fitted 1250 times to the discovery sample containing N=1250 individuals, each time leaving out one individual from the sample. Hence, 1250 clustering solutions were generated for each analyzed K value. The cluster solution where the parameter K reached the best median ICL value in the resampling procedure was selected as the final model (K=5).

The final assignment of individuals to clusters for the optimal model AkBkQkDk and parameter K=5 was performed based on the 1250 leave-one-out cluster solutions by using the majority voting scheme. We conducted majority voting using the function *majority_voting*, as implemented in the *R* package *dicer* [23]. Here, we set the argument *is.relabelled = False*, *i.e.*, the data was relabeled using the first clustering iteration as the reference.

The stability of the clustering solution was assessed with an additional 100 runs of the model-fitting procedure, each time using 95% of the resampled discovery sample (Suppl. Fig. S4C). The thus obtained solution was compared to the previously determined optimal solution using the Rand and Jaccard similarity indices. Note that the 5% holdout for the stability analysis was chosen to generate an additional view of the solution, using the different holdout proportion of 1/20, in-between the 1/5 and 1/1250 employed for the model and K choice, respectively.

We used three different resampling strategies in the different steps to reduce the risk of overfitting, which is higher when employing only one approach.

The illustration of the complete clustering workflow is shown in Figure S3, while the results of each step provided in Figure S4. For a list of variables used in the clustering, see Table S2; for a list of variables used for the post-hoc characterization of clusters, see Table S3.

Methods S5. Characterization of clusters

High-dimensional discriminant analysis

Supervised high-dimensional discriminant analysis (HDDA) [20, 24] is a subspace classification algorithm that incorporates the same Gaussian mixture model fitting for the high-dimensional data as the corresponding unsupervised HDDC does.

Note that because HDDA incorporates the same modeling framework as HDDC, it allows for applying the HDDC model (AKBKQKDK) in the analysis of the importance of variables. In this analysis, the same 57 variables and the best-fitting model identified by HDDC (AkBkQkDk, K=5) were used to predict the cluster labels. The prediction model was run 100 times on the discovery sample, each time on a separate random 70% *vs.* 30% split of training and test data, respectively. For each run, the area under the receiver-operator curve (AUC) was calculated using the *R* package *pROC* [25]. The average metrics across the 100 runs were used as the final measure of classification success.

The importance of variables was assessed to identify the relevant variables for distinguishing each cluster from the others. This importance was measured by calculating the decrease in the model's AUC metric after the values of the respective variable were randomly permuted. The larger the drop of AUC after permutation, the more relevant the variable. The variables were ranked using the average AUC drop after 100 runs.

Lasso regularized regression

Lasso (least absolute shrinkage and selection operator) regularized regression [26] analyses were conducted using the *R* package *glmnet* [27], with 24 predictors (10 PGSs, 4 self-reported family history variables, 8 ancestry components, age, and gender). Lasso penalizes regression coefficients and may shrink them to zero; the degree of penalization depends on the parameter λ. To tune λ, the discovery sample was split 1000 times into 70% training and 30% test data, using stratification based on cluster labels. On each of the 1000 training sets, λ was tuned via three-fold cross-validation. In each run, the λ minimizing the cross-validation error, *i.e.*, maximizing the AUC (λ_{min}), was used to obtain Lasso coefficients on the test set. AUC, sensitivity, and specificity were calculated in each run; the average metrics of all runs were reported. The cutoff for the sensitivity and specificity was determined using the *MaxSpSe* method of the *OptimalCutpoints* package [28]. Lastly, to get the final model, *i.e.*, variable sets and their corresponding coefficients for the cluster prediction, lasso was fit to the full dataset with λ equal to the median value of all λ_{min} chosen during the tuning procedure.

Methods S6. Statistical testing

The Westfall and Young method for significance testing controls the family-wise error rate while accounting for the possible dependence structure of the analyzed variables. The adjusted *p*-values were directly estimated via 20,000 permutations, using the *mt.minP* function from the Bioconductor *multtest* package [29] with Welch's t statistic. After adjusting the *p*-values using the Westfall and Young procedure, they were, subsequently, also corrected for the number of comparisons made using Bonferroni's method. This number of comparisons corresponded to the number of clusters in the *one-vs-all* analyses and to *(K*(K-1))/2* for *one-vs-one* (pairwise) analyses.

Methods S7. Assessment of the information gain by PGS

For the assessment of the information gain of PGSs in the prediction of clusters, we analyzed four multinomial regression models using the *R* package VGAM [30] with cluster labels as the dependent variable and the following predictors: a) PGS and ancestry components (ACs), b) family history only, c) family history and ACs, d) the full model with PGS, family history, and ACs. Age and gender were used as covariates in all four models. The model performance was assessed using Nagelkerke's pseudo-R^2, the Akaike information criterion (AIC), and likelihood-ratio tests. For the calculation of the pseudo-R^2, a null model containing only the covariates age and gender was used.

Methods S8. Replication analysis
To identify the discovery-stage clusters in the replication data, each of the binary discovery-stage *one-vs-all* HDDA classification models was used for *one-vs-all* predictions in the replication dataset, thereby conducting K^2 comparisons. The discovery-stage model producing the best prediction above a threshold of 70% was chosen to assign the cluster identity in the replication sample.
After matching the discovery and replication clusters, further statistical analysis, *i.e.*, significance testing with the Westfall and Young procedure (described in the Methods S6) was carried out to confirm the matching and homogeneity of linked discovery and replication clusters. As for the discovery sample, *p*-values adjusted by the Westfall and Young procedure were further corrected using Bonferroni's method for the five comparisons made.
We analyzed the performance of the discovery-stage Lasso regression models of genetic variables in the replication sample by using the λ model parameter, as optimized in the discovery sample (see Methods S5), for a Lasso regression analysis of the previously matched replication clusters.

Results S1. Follow-up analyses of the severity continuum
We conducted a principal component analysis (PCA) to assess whether a simple severity component can explain our clusters. The first component (PC1) of the PCA performed on all variables explained 45.5% of the variance. The SCL90R global severity index was the top-contributing variable for this component (Table S21). This PC1 correlated with the cluster labels (Spearman ρ=0.75). However, it predominantly captured the distinction between health and disease instead of the overall severity gradient: When removing cluster 0 and only correlating clusters 1-4 with PC1, the correlation dropped to ρ=0.38.
We applied *SigClust* [31] to explore the hypothesis further that a severity continuum might best explain our data. However, this method did not find statistical support for the data coming from a single continuous Gaussian distribution – when analyzing all data, only psychiatric patients (excluding healthy controls), or only the more severely affected clusters 1-4 (each analysis: $p \leq 0.001$). The finding that a simple severity continuum does not accurately represent our data corroborates the hypothesis that we identified five categorical clusters ranked along but not exactly corresponding to a severity continuum. The subspace-specific HDDC algorithm, optimized for handling high-dimensional and correlated features, thus produced clusters capturing more than a severity continuum. Notably, also a bimodal distribution with two subgroups (e.g., psychiatric patients and healthy controls) did not model our data accurately. Models with K=2 clusters achieved the poorest fits in all steps of our clustering pipeline (see Suppl. Figure S4).

Results S2. Assessment of the variable selection on the clustering solution
For obtaining a complete picture of symptom patterns, we considered all available data, including clinician rating scales and self-report questionnaires. While the total symptom spectrum was assessed with the general self-report questionnaire SCL-90R, we additionally included dedicated self-report questionnaires for depression (BDI-II) and anxiety (STAI) symptoms, given their frequent use in clinical practice. While the two additional questionnaires highly correlate with the respective SCL-90R symptom scales (Spearman ρ=0.86 and ρ=0.71, respectively; see Fig. S1), suggesting no overrepresentation of depression and anxiety symptoms, we cannot entirely exclude that the variable selection influenced the obtained clustering solution.
In addition, we did not balance for age or sex when recruiting the diagnostic subsamples due to the chosen recruitment approach of generating a representative clinical sample from the referrals of the participating hospitals. This approach led to age differences between diagnostic groups, also resulting in age differences also between clusters. To control for these differences, age was considered as a control variable in all supervised cluster comparisons.

Table S1. Characterization of the replication sample and clusters

We compared age, sex, and diagnosis of the discovery and replication datasets. Age and diagnosis differed significantly (*p*=0.01 and *p*=0.002, respectively), while sex did not (*p*=0.16). Proportions of single diagnostic categories were significant for healthy controls (p=0.005) and MDD (p-value 0.003). Other categories did not show significant differences (BD, *p*=0.4; SCZ, *p*=0.1; SZA, *p*=1; Other diagnosis, *p*=0.07). Age at Onset was defined using OPCRIT item 4, it was not available for HC.
Abbreviations and clarifications: BMI = Body mass index; Family history Any = Family history of any psychiatric disorder; AAO = Age at Onset (*not available for healthy controls); HC = Healthy controls; BD = Bipolar Disorder; MDD = Major Depressive Disorder; SCZ = Schizophrenia; SZA = Schizoaffective Disorder; Other = other psychiatric diagnoses including anxiety, adjustment, and substance use disorders; SANS/SAPS = Scale for the Assessment of Negative/Positive Symptoms; SF36; 36-Item Short Form Survey.
Variable ranges and interpretations: Quality of life [0-100, 0=disability]; HAMD 21 item [0-66, 0=not present]; HAMA [0-56, 0=not present]; BDI [0-63, 0=not present]; SAPS/SANS [0-86/0-80, 0=not present]; CTQ [5-25, 5=less severe experiences].

Please see the separate Excel file for the table.

Table S2. Variables used in the clustering procedure

The baseline variables listed in the table below were used for clustering. The values in this table represent the mean (SD) of the 57 variables used in the clustering procedure for the discovery (N=1250) and replication (N=622) sample.

Note that some of the available baseline variables were not included in the clustering procedure. These variables are displayed in Table S3.
Most of these variables were excluded from the clustering analysis because they were confounded with diagnostic groups or strongly differentiated psychiatric patients from healthy controls. Medication, hospitalization, lifestyle, and sociodemographic variables were in this group.
To avoid over-representing specific diagnostic aspects, we used the SANS and SAPS variables as sum scores for clustering. We present their subscales in Table S3. For the Childhood Trauma Questionnaire (CTQ), we used the five subscales but not the sum score for clustering.
The Global Assessment of Functioning (GAF) was excluded from the clustering since the SF36 and SCL90R variables cover the aspects of severity and day-to-day functioning. Furthermore, GAF had higher missingness than these. The age at onset was not available for healthy controls and thus not used for clustering.
Two variables were completely excluded from all analyses and are not shown in Table S3:
We completely excluded the Trauma History Questionnaire (THQ) from our analyses, given its high correlation with the Life Events Questionnaire (LEQ). The latter LEQ was included for clustering.
Finally, we excluded the Life chart, which is an instrument to represent disease episodes graphically and does not provide quantitative scores.

Please see the separate Excel file for the table.

Table S3. Characterization of the discovery sample with variables not used for clustering

The baseline variables listed below were available for the present study but not directly used in the clustering. Instead, they were used for the *post-hoc* characterization of the clusters. For an explanation of why these variables were not used for clustering, see the legend of Table S2 above.
*The age at onset (AAO) was not available for healthy controls.

Please see the separate Excel file for the table.

Table S4. Site-specific differences across clusters and diagnoses

The percentage was calculated from the corresponding total (for example Cluster 0, HC Marburg: 278/448 = 0.62).
Significant site-specific differences were observed for cluster 0 ($p = 0.0003$) and cluster 4 ($p = 0.008$), as well as for other diagnoses ($p = 0.0002$). Note that both sites differed in their recruitment strategies: The Münster site focused on recruiting depressive patients, while the majority of psychotic patients was acquired in Marburg. Healthy subjects were recruited via newspaper advertisements. In addition, recruitment in Münster started nine months after Marburg. Differences between sites in clusters 0 and 4 are likely due to these different recruitment strategies, given that cluster 0 consists mainly of healthy controls and cluster 4 contains most of the psychotic patients.
Abbreviations: HC = Healthy controls; BD = Bipolar Disorder; MDD = Major Depressive Disorder; SCZ = Schizophrenia; SZA = Schizoaffective Disorder; Other = other psychiatric diagnoses including anxiety, adjustment, and substance use disorders

			**Diagnosis**					
**Cluster**	**Site**	**Total**	**HC**	**BD**	**MDD**	**SCZ**	**SZA**	**Other**
Cluster 0	Total	535	448	9	56	4	2	16
	Marburg	317 (59%)	278 (62%)	7 (78%)	27 (48%)	1 (25%)	1 (50%)	3 (19%)
	Münster	218 (41%)	170 (38%)	2 (22%)	29 (52%)	3 (75%)	1 (50%)	13 (81%)
Cluster 1	Total	38	19	1	17	1	0	0
	Marburg	26 (68%)	15 (79%)	0 (0%)	11(65%)	0 (0%)	0 (0%)	0 (0%)
	Münster	12 (32%)	4 (21%)	1 (100%)	6 (35%)	1(100%)	0 (0%)	0 (0%)
Cluster 2	Total	266	78	15	152	8	5	8
	Marburg	182 (68%)	54 (69%)	10 (67%)	104(68%)	7 (88%)	5(100%)	2 (25%)
	Münster	84 (32%)	24 (31%)	5 (33%)	48 (32%)	1 (13%)	0 (0%)	6 (75%)
Cluster 3	Total	215	34	26	147	3	2	3
	Marburg	143 (67%)	23 (68%)	20 (77%)	98 (67%)	1 (33%)	1 (50%)	0 (0%)
	Münster	72 (33%)	11 (32%)	6 (23%)	49 (33%)	2 (67%)	1 (50%)	3 (100%)
Cluster 4	Total	196	11	24	105	37	16	3
	Marburg	144 (73%)	11 (100%)	13 (54%)	76 (72%)	32(86%)	9 (56%)	3 (100%)
	Münster	52 (27%)	0 (0%)	11 (46%)	29 (28%)	5 (14%)	7 (44%)	0 (0%)
Total	Total	1250	590	75	477	53	25	30
	Marburg	812 (65%)	381 (65%)	50 (67%)	316(66%)	41(77%)	16(64%)	8 (27%)
	Münster	438 (35%)	209 (35%)	25 (33%)	161(34%)	12(23%)	9 (36%)	22 (73%)

Table S5. Most important features from the HDDA analysis

The most important variables for each cluster from the '*one-vs.-all'* HDDA classification analysis. The importance was calculated based on the average AUC drop, as explained in the Methods S5. The numbers in the parentheses represent the mean AUC drop across 100 iterations, with the standard error (SE) provided in square brackets. The numbers are in the [-1,1] range. Therefore, an average of -0.01 represents a 1 percentage point (p.p.) drop of the overall AUC. Plots of these variables per cluster are shown in Figure S5.

Cluster 0 (mean [SE])	Cluster 1 (mean [SE])	Cluster 2 (mean [SE])	Cluster 3 (mean [SE])	Cluster 4 (mean [SE])
CTQ Sexual abuse (-0.01 [0.0003])	SF36 Bodily pain (-0.08 [0.0124])	Maternal bonding (-0.016 [0.0012])	SF36 Role physical (-0.014 [0.0014])	SAPS (Positive symptoms) (-0.018 [0.0008])
LEQ Negative Events score (-0.009 [0.0003])	SF36 Role physical (-0.043 [0.0125])	CTQ Emotional neglect (-0.015 [0.0012])	CTQ Emotional neglect (-0.011 [0.0015])	YMRS (-0.013 [0.0007])
SAPS (Positive Symptoms) (-0.009 [0.0003])	NEO-FFI Agreeableness (-0.021 [0.0129])	Paternal bonding (-0.014 [0.0012])	Maternal bonding (-0.011 [0.0015])	CTQ Physical abuse (-0.011 [0.0005])
CTQ Physical abuse (-0.008 [0.0002])	RSQ Lack of trust (-0.018 [0.0131])	SF36 Physical functioning (-0.013 [0.0011])	CTQ Emotional abuse (-0.01 [0.0015])	CTQ Sexual abuse (-0.01 [0.0005])
SF36 Physical functioning (-0.008 [0.0003])	Maternal bonding (-0.017 [0.013])	SF36 Role physical (-0.009 [0.0011])	CTQ Physical neglect (-0.007 [0.0014])	LEQ Negative Events score (-0.006 [0.0005])
YMRS (-0.008 [0.0003])	STAIS (-0.017 [0.013])	ACE Sum (-0.009 [0.0012])	ACE Sum (-0.006 [0.0014])	SANS (Negative symptoms) (-0.005 [0.0004])
SANS (Negative symptoms) (-0.007 [0.0002])	SHAPS (Anhedonia) (-0.013 [0.0127])	RSQ Avoidance of closeness (-0.009 [0.0011])	SF36 Physical functioning (-0.006 [0.0014])	CTQ Physical neglect (-0.004 [0.0004])
SF36 Role physical (-0.007 [0.0002])	SF36 Physical functioning (-0.012 [0.013])	CTQ Emotional abuse (-0.008 [0.0012])	CTQ Physical abuse (-0.006 [0.0014])	LEQ Positive Events score (-0.003 [0.0004])
SCL90R Phobic anxiety (-0.007 [0.0002])	NEO-FFI Neuroticism (-0.012 [0.013])	CTQ Physical abuse (-0.007 [0.0011])	SF36 Energy (-0.005 [0.0014])	VLMT Sum (-0.002 [0.0004])
CTQ Physical neglect (-0.006 [0.0002])	SAPS (Positive symptoms) (-0.01 [0.0128])	SAPS (Positive Symptoms) (-0.007 [0.0011])	Paternal bonding (-0.005 [0.0014])	CTQ Emotional abuse (-0.002 [0.0004])

Table S6. Lasso - clinical variables average coefficients
The table shows the mean (SEM) of coefficients across 1000 iterations. Variables are sorted based on the strongest absolute effect sizes for the model Cluster 0 *vs.* all. The top ten variables for each cluster, based on the absolute mean coefficient, are indicated in **bold** font.

Please see the separate Excel file for the table.

Table S7. Characterization of the discovery sample and its clusters regarding variables used in the clustering process

Please see the separate Excel file for the table.

Table S8. MDD subtype analysis

This table shows phenotypic characteristics of MDD patients, constituting a secondary, descriptive analysis to assess the heterogeneity within MDD patients. The significance of genetic variables was tested using the Westfall and Young procedure (Methods S6). Here, only the significant variables are shown. The ADHD PGS (*) was not significant in the significance analysis of the full sample including all diagnoses. The distributions of the two significant PGS per cluster are shown in Figure S6 A-B. The age at onset was defined according to OPCRIT item 4.
Abbreviations: EA, educational attainment; ADHD, Attention deficit/hyperactivity disorder, W-Y, Westfall and Young.

Please see the separate Excel file for the table.

Table S9. *One-vs-all* HDDA classification analysis using only MDD-diagnosed patients

This table shows the most important clinical variables for each cluster when analyzing only MDD patients. The variables were identified by the *one-vs.-all* HDDA classification analysis using n=477 MDD patients and their respective cluster labels. The importance was calculated based on the average AUC drop, as explained in the Methods S5.
The numbers in the parentheses represent the average (SE) drop across 100 iterations. The numbers are in [-1,1] range, therefore an average of -0.01 represents a 1 percentage point (p.p.) drop of the overall AUC.

Cluster 0 (mean [SE])	Cluster 1 (mean [SE])	Cluster 2 (mean [SE])	Cluster 3 (mean [SE])	Cluster 4 (mean [SE])
CTQ Sexual abuse (-0.014 [0.0008])	Maternal bonding (0.023 [0.0128])	SF36 Physical functioning (-0.014 [0.0017])	Maternal bonding (-0.013 [0.0022])	CTQ Physical abuse (-0.03 [0.0013])
SAPS (Positive symptoms) (-0.013 [0.0008])	SF36 Bodily pain (0.033 [0.0129])	SF36 Role physical (-0.013 [0.0016])	CTQ Emotional neglect (-0.012 [0.0022])	CTQ Sexual abuse (-0.022 [0.0011])
CTQ Physical neglect (-0.012 [0.0008])	SF36 Role physical (0.037 [0.0129])	CTQ Physical abuse (-0.011 [0.0017])	CTQ Emotional abuse (-0.011 [0.0021])	SAPS (Positive Symptoms) (-0.02 [0.0011])
LEQ Negative Events score (-0.012 [0.0007])	YMRS (0.053 [0.0127])	CTQ Emotional neglect (-0.008 [0.0017])	SF36 Role Physical (-0.01 [0.0021])	CTQ Emotional abuse (-0.018 [0.0012])
CTQ Physical abuse (-0.011 [0.0007])	SF36 Energy (0.054 [0.0129])	RSQ Avoidance of closeness (-0.007 [0.0017])	CTQ Physical abuse (-0.007 [0.002])	ACE Sum (-0.017 [0.0011])
ACE Sum (-0.011 [0.0007])	SHAPS (0.056 [0.013])	CTQ Emotional abuse (-0.006 [0.0016])	CTQ Physical neglect (-0.007 [0.0021])	LEQ Positive Events score (-0.016 [0.0011])
SCL Positive symptoms (-0.011 [0.0007])	Social support (0.057 [0.0132])	ACE Sum (-0.006 [0.0017])	ACE Sum (-0.007 [0.0021])	SANS (Negative symptoms) (-0.015 [0.001])
SF36 Social functioning (-0.011 [0.0007])	Verbal IQ (0.058 [0.0128])	Maternal bonding (-0.006 [0.0017])	SF36 Bodily pain (-0.006 [0.0021])	SCL90R Phobic fear (-0.015 [0.001])
SANS (Negative symptoms) (-0.01 [0.0007])	SAPS (Positive symptoms) (0.058 [0.0127]	CTQ Sexual abuse (-0.006 [0.0017])	SF36 General health (-0.005 [0.0021])	VLMT Sum (-0.015 [0.0011])
SF36 Mental health (-0.01 [0.0008])	CTQ Emotional neglect (0.06 [0.0131])	SCL90R Somatization (-0.006 [0.0016])	SF36 Role emotional (-0.005 [0.002])	CTQ Physical neglect (-0.014 [0.0009])

Table S10. Metrics of genetic lasso regularized regression prediction models

The table shows the results of lasso regularized regression models applied to characterize clusters using genetic variables. The model consisted of 24 predictors – ten PGS, four family history variables, eight ancestry components, age, and gender, and was applied both in *one-vs-all* and *one-vs-one* analyses. In the one-*vs.*-one comparisons, the cluster tagged as control [28], used for calculation of the sensitivity and specificity, was the respective cluster with the higher rank number. The calculation of all metrics is explained in Methods S5.
An AUC between 50% and 60% is considered random model performance. The table lists models with an AUC >60%, *i.e.*, showing above-random performance.
Performance metrics of the models describing the two extreme clusters (cluster 0 *vs.* all, cluster 4 *vs.* all, and cluster 0 *vs.* cluster 4) were highest.

Model	AUC	Sensitivity	Specificity
Cluster 0 *vs.* all	71%	66%	66%
Cluster 4 *vs.* all	73%	67%	67%
Cluster 0 *vs.* Cluster 4	81%	75%	75%
Cluster 0 *vs.* Cluster 2	67%	67%	63%
Cluster 2 *vs.* Cluster 4	67%	64%	62%
Cluster 0 *vs.* Cluster 3	66%	63%	63%
Cluster 3 *vs.* Cluster 4	64%	61%	60%

Table S11. Genetic lasso regularized regression prediction models: summary statistics
The table shows summary statistics from 1000 runs of lasso regularized regression models applied to characterize clusters using genetic variables (see Methods S5). The numbers in the table represent the number of times the variable had a non-zero coefficient in 1000 runs and, in parentheses, the mean coefficient calculated from 1000 runs. The standard error of the mean is shown in square brackets.

Please see the separate Excel file for the table.

Table S12. Significance testing of genetic analyses with the Westfall and Young procedure – *one-vs-all* comparisons

This table shows the results of *one-vs-all* significance testing of genetic analyses with the Westfall and Young procedure (Methods S6). As in all genetic analyses, 24 variables (ten PGS, four family history variables, eight ancestry components, age, and gender) were used to test for significant differences. Only the comparisons with significant variables after the Westfall and Young adjustment are shown. The distribution of significant PGS per cluster is shown in Figure 1 E-H.
n.s.: adjusted $p>0.05$; Any = any assessed psychiatric disorder (MDD, BD, SCZ/SZA).

One-*vs*-All Comparison	Variable	*t*-statistic	*p*-value adjusted using Westfall and Young	*p*-value further adjusted for the number of comparisons (N=5)
Cluster 0 *vs.* all	Age	-7.9	8×10^{-4}	4×10^{-3}
	Family History Any	-10.2	8×10^{-4}	4×10^{-3}
	Family History BD	-4.2	8×10^{-4}	4×10^{-3}
	Family History MDD	-7.0	8×10^{-4}	4×10^{-3}
	PGS Cross psychiatric disorder	-4.8	8×10^{-4}	4×10^{-3}
	PGS MDD	-4.8	1×10^{-3}	8×10^{-3}
	PGS Schizophrenia	-3.6	9×10^{-3}	4×10^{-2}
	PGS Educational attainment	3.3	2×10^{-2}	9×10^{-2} (n.s.)
	PGS Neuroticism	-3.1	3×10^{-2}	1×10^{-1} (n.s.)
Cluster 2 *vs.* all	Family History Any	4.6	1×10^{-3}	5×10^{-3}
	Family History MDD	3.9	6×10^{-3}	3×10^{-2}
Cluster 4 *vs.* all	Age	6.1	9×10^{-4}	4×10^{-3}
	Family History Any	6.4	9×10^{-4}	4×10^{-3}
	PGS Cross psychiatric disorder	4.0	3×10^{-3}	1×10^{-2}
	PGS MDD	3.5	7×10^{-3}	4×10^{-2}
	PGS Schizophrenia	3.8	3×10^{-3}	1×10^{-2}
	PGS Educational attainment	-4.3	9×10^{-4}	4×10^{-3}

Table S13. Significance testing of genetic analyses with the Westfall and Young procedure – *one-vs-one* comparisons

This table shows the results of *one-vs-one* significance testing of genetic analyses with the Westfall and Young procedure (Methods S6). As in all genetic analyses, 24 variables (ten PGS, four family history variables, eight ancestry components, age, and gender) were used to test for significant differences. Only the comparisons with significant variables after the Westfall and Young adjustment are shown.
The distribution of significant PGS per cluster is shown in Figure 1 E-H.
n.s.: adjusted p>0.05; Any = any assessed psychiatric disorder (MDD, BD, SCZ/SZA).

One-*vs*-One Comparison	Variable	*t*-statistic	*p*-value adjusted using Westfall and Young	*p*-value further adjusted for the number of comparisons (N=10)
Cluster 0 *vs.* Cluster 2	Age	-3.9	5×10^{-3}	5×10^{-2}
	Family History Any	-7.7	1×10^{-3}	1×10^{-2}
	Family History MDD	-5.8	1×10^{-3}	1×10^{-2}
	PGS Cross psychiatric disorder	-3.6	6×10^{-3}	6×10^{-2} (n.s.)
	PGS MDD	-3.5	1×10^{-2}	1×10^{-1} (n.s.)
	PGS Neuroticism	-3.4	1×10^{-2}	1×10^{-1} (n.s.)
Cluster 0 *vs.* Cluster 3	Age	-5.2	1×10^{-3}	1×10^{-2}
	Family History Any	-5.3	1×10^{-3}	1×10^{-2}
	Family History MDD	-4.3	1×10^{-3}	1×10^{-2}
Cluster 0 *vs.* Cluster 4	Age	-7.9	8×10^{-4}	8×10^{-3}
	AC4	3.1	3×10^{-2}	3×10^{-1} (n.s.)
	Family History Any	-9.1	8×10^{-4}	8×10^{-3}
	Family History BD	-4.2	1×10^{-2}	1×10^{-1} (n.s.)
	Family History MDD	-4.4	1×10^{-3}	1×10^{-2}
	PGS Cross psychiatric disorder	-5.0	8×10^{-4}	8×10^{-3}
	PGS Schizophrenia	-4.4	8×10^{-4}	8×10^{-3}
	PGS Educational attainment	4.7	8×10^{-4}	8×10^{-3}
Cluster 2 *vs.* Cluster 4	Age	-3.9	4×10^{-3}	4×10^{-2}
	Family History BD	-3.3	2×10^{-2}	2×10^{-1} (n.s.)
Cluster 3 *vs.* Cluster 4	Family History BD	-3.1	4×10^{-2}	4×10^{-1} (n.s.)
	PGS Educational attainment	3.2	3×10^{-2}	3×10^{-1} (n.s.)

Table S14. Genetic analysis with diagnostic (DSM-IV) labels

A) Westfall and Young significance testing

One-*vs*-All Comparison	Variable	*t*-statistic	*p*-value adjusted using Westfall and Young	*p*-value further adjusted for the number of comparisons **(N=6)**
HC *vs.* all	Age	-7.0	2×10^{-3}	1×10^{-2}
	Family History Any	-10.7	2×10^{-3}	1×10^{-2}
	Family History MDD	-7.5	2×10^{-3}	1×10^{-2}
	Family History BD	-5.2	2×10^{-3}	1×10^{-2}
	PGS CD	-5.1	2×10^{-3}	1×10^{-2}
	PGS BD	-4.1	2×10^{-3}	1×10^{-2}
	PGS MDD	-5.8	2×10^{-3}	1×10^{-2}
	PGS SCZ	-4.7	2×10^{-3}	1×10^{-2}
BD *vs.* all	Age	6.2	2×10^{-3}	1×10^{-2}
MDD *vs.* all	Age	3.4	1×10^{-2}	7×10^{-2} (n.s)
	Family History	9.2	2×10^{-3}	1×10^{-2}
	Family History MDD	7.9	2×10^{-3}	1×10^{-2}
	PGS CD	3.1	2×10^{-2}	1×10^{-1} (n.s)
	PGS MDD	5.8	2×10^{-3}	1×10^{-2}
	PGS Neuroticism	4.1	2×10^{-3}	1×10^{-2}
SCZ vs. all	PGS SCZ	4.2	4×10^{-3}	2×10^{-2}

B) Lasso with diagnostic labels – One *vs.* all models metrics

The table shows the results of lasso regularized regression models applied to characterize diagnostic (DSM) categories using genetic variables. The model consisted of 24 predictors – ten PGS, four family history variables, eight ancestry components, age, and gender, and was applied both in *one-vs-all* analyses.

Model	AUC	Sensitivity	Specificity
HC vs. all	72%	67%	67%
MDD vs. all	68%	64%	64%
BD vs. all	71%	67%	65%
SCZ vs. all	67%	64%	62%
SZA vs. all	58%	61%	64%
Other vs. all	53%	61%	48%

C) Lasso with diagnostic labels – Summary statistics from 1000 runs

The table shows number of times variable had non-zero coefficient across 1000 runs and average (SEM) coefficient calculated from 1000 runs.
Values in the table: number of occurrences (mean coefficient (SEM)). Maximum number of occurrences: 1000.

Please see the separate Excel file for the table.

D) Assessment of the PGS information gain
The table shows the results of multinomial regression analyses for the assessment of the information gain by PGS in the prediction of clusters, as explained in Methods S7.
Nagelkerke's pseudo-R^2 was calculated using the following formula:
(1 – exp((2/N) * (logLik(null_model) – logLik(full_model)))) / (1 – exp(logLik(null_model))^(2/N)).
For the column ‘Nagelkerke's pseudo-R^2‘, the intercept model was used as the null model in the above formula. Thus, this column shows the variance explained by the row-specific model.
For the column ‘Increase in Nagelkerke's pseudo-R^2‘, the null model was the null model shown in row 1, containing only the covariates age and gender. Thus, this column shows the increase in the variance explained of models A-D over the null model.
Abbreviations: AIC = Akaike information criterion; Y = outcome variable (cluster labels); PGSs = all ten polygenic scores (see Methods S3); Family History = four self-reported family history variables (any psychiatric disorder, MDD, BD, SCA/SCZ); Acs = eight ancestry components; n.s. = not significant (adjusted p>0.05).

**Model**	**Regression model**	**AIC**	**Nagelkerke's pseudo-R^2**	**Increase in Nagelkerke's pseudo-R^2**	**Likelihood ratio test (LRT)**	**LRT *p*-value**
**Null model**	Y ~ Age + Gender	2657.3	7.49%		Null model *vs.* Model A	1×10^{-7}
					Null model vs. Model D	1×10^{-20}
**Model A**	Y ~ Age + Gender + PGSs + ACs	2661.8	20.73%	14.30%	Model A *vs.* Model D	2×10^{-18}
**Model B**	Y ~ Age + Gender + Family History	2544.9	19.09%	12.54%	Model B v*s.* Model D	2×10^{-5}
**Model C**	Y ~ Age + Gender + Family History + ACs	2584.1	21.95%	15.63%	Model B *vs.* Model C	0.4 (*n.s.*)
**Model D**	Y ~ Age + Gender + Family History + PGSs + ACs (*full model*)	2570.1	29.39%	23.67%	Model C *vs.* Model D	6×10^{-7}

Table S15. Assessment of the PGS information gain (cluster labels)

The table shows the results of multinomial regression analyses for the assessment of the information gain by PGS in the prediction of clusters, as explained in Methods S7.
Nagelkerke’s pseudo-R^2 was calculated using the following formula:
(1 – exp((2/N) * (logLik(null_model) – logLik(full_model)))) / (1 – exp(logLik(null_model))^(2/N)).
For the column ‘Nagelkerke’s pseudo-R^2‘, the intercept model was used as the null model in the above formula. Thus, this column shows the variance explained by row-specific model.
For the column ‘Increase in Nagelkerke’s pseudo-R^2‘, the null model was the null model shown in row 1, containing only the covariates age and gender. Thus, this column shows the increase in the variance explained of models A-D over the null model.
Abbreviations: AIC = Akaike information criterion; Y = outcome variable (cluster labels); PGSs = all ten polygenic scores (see Methods S3); Family History = four self-reported family history variables (any psychiatric disorder, MDD, BD, SCA/SCZ); Acs = eight ancestry components; n.s. = not significant (adjusted p>0.05).

**Model**	**Regression model**	**AIC**	**Nagelkerke’s pseudo-R^2**	**Increase in Nagelkerke’s pseudo-R^2**	**Likelihood ratio test (LRT)**	**LRT *p*-value**
**Null model**	Y ~ Age + Gender	3073.1	7.12%		Null model *vs.* Model A	2×10^{-6}
					Null model vs. Model D	2×10^{-18}
**Model A**	Y ~ Age + Gender + PGSs + ACs	3076.2	17.94%	11.70%	Model A *vs.* Model D	2×10^{-17}
**Model B**	Y ~ Age + Gender + Family History	2975.5	17.12%	10.77%	Model B v*s.* Model D	5×10^{-5}
**Model C**	Y ~ Age + Gender + Family History + ACs	2999.1	20.01%	13.89%	Model B *vs.* Model C	0.1 (*n.s.*)
**Model D**	Y ~ Age + Gender + Family History + PGSs + ACs (*full model*)	2991.4	25.95%	20.28%	Model C *vs.* Model D	2×10^{-5}

Table S16. Significance testing with the Westfall and Young procedure – comparison of discovery-replication pairs

After the discovery and replication clusters were matched, further statistical analysis, *i.e.*, significance testing with the Westfall and Young procedure (described in the Supplementary Methods S6), was carried out to confirm the matching and homogeneity of linked discovery and replication clusters. All 57 variables which entered the clustering process were tested. The table shows only the variables that were significantly different between the linked clusters.
A matching cluster assignment was confirmed for four clusters. n.s.: adjusted p>0.05.

Discovery-Replication cluster pair	Significant variable	t- statistic	*p*-value adjusted using Westfall and Young	*p*-value further adjusted for the number of comparisons (N=5)
Cluster 0 – Cluster 0	SF36 General health	5.7	2×10^{-3}	1×10^{-1}
	NEO-FFI Agreeableness	4.2	4×10^{-3}	2×10^{-2}
	Letter Number Span test	4.1	4×10^{-3}	2×10^{-2}
	Paternal Bonding	3.5	3×10^{-2}	1×10^{-1} (n.s.)
	CTQ Physical Neglect	-3.8	4×10^{-2}	2×10^{-1} (n.s.)
Cluster 2 – Cluster 2	NEO-FFI Extraversion	-4.1	4×10^{-3}	2×10^{-2}
	NEO-FFI Neuroticism	3.9	1×10^{-2}	5.7×10^{-1} (n.s.)
	CTQ Emotional Neglect	3.8	2×10^{-2}	8×10^{-2} (n.s.)
	RS25 Sum Score	-3.8	2×10^{-2}	8×10^{-2} (n.s.)
	RSQ Avoidance of closeness	3.4	4×10^{-2}	2×10^{-1} (n.s.)
	RSQ Lack of trust	3.4	4×10^{-2}	2×10^{-1} (n.s.)
Cluster 3 – Cluster 3	SCL90R Paranoid ideation	-4.1	4×10^{-3}	2×10^{-2}
	SCL90R Somatization	-3.9	6×10^{-3}	3×10^{-2}
	SCL90R Psychoticism	-3.8	6×10^{-3}	3×10^{-2}
	SCL90R Global severity index	-3.9	1×10^{-2}	8×10^{-2} (n.s.)
	SF36 Physical functioning	3.5	2×10^{-2}	1×10^{-1} (n.s.)
	SCL90R Interpersonal sensitivity	-3.3	4×10^{-2}	2×10^{-1} (n.s.)
Cluster 4 – Cluster 4	CTQ Physical Neglect	-3.8	7×10^{-3}	3×10^{-2}
	SAPS	-4.5	9×10^{-3}	4×10^{-2}
	SANS	-3.7	1.5×10^{-2}	7×10^{-2} (n.s.)

Table S17. Characterization of the replication sample and its clusters with variables used in the clustering process

Please see the separate Excel file for the table.

Table S18. Metrics of genetic lasso regularized regression models in the replication sample

After the discovery and replication clusters had been matched, the Lasso model parameters trained on the discovery sample were used for lasso regularized regression analyses of the genetic variables in the replication sample (see Methods S8).
An AUC between 50% and 60% is considered random model performance. The table lists models with an AUC >60%, *i.e.*, showing above-random performance.
Performance metrics of the models describing the two extreme clusters (cluster 0 *vs.* all, cluster 4 *vs.* all, and cluster 0 *vs.* cluster 4) were highest, similar to the discovery-stage analysis (Table S10).

**Model**	**AUC**	**Sensitivity**	**Specificity**
Cluster 0 *vs.* all	63%	60%	60%
Cluster 4 *vs.* all	68%	67%	66%
Cluster 0 *vs.* Cluster 4	75%	72%	72%
Cluster 2 *vs.* Cluster 4	69%	72%	67%
Cluster 3 *vs.* Cluster 4	61%	60%	60%
Cluster 0 *vs.* Cluster 2	60%	59%	59%
Cluster 0 *vs.* Cluster 3	60%	70%	53%

Table S19. Replication – Significance testing with the WY procedure, *one-vs-all* comparisons

Only the comparisons with significant variables after the first adjustment are shown.
n.s.: not significant at an α=0.05 threshold. WY = Westfall and Young.

One-*vs*-All Comparison	Variable	t-statistic	*p*-value adjusted using Westfall and Young	*p*-value further adjusted for the number of comparisons (N=5)
Cluster 0 *vs.* all	Age	-3.9	3×10^{-2}	1×10^{-1} (n.s.)
	PGS Cross psychiatric disorder	-3.7	7×10^{-3}	3×10^{-2}
	PGS Schizophrenia	-3.2	1×10^{-3}	5×10^{-3}
Cluster 2 *vs.* all	Age	-3.8	1×10^{-2}	5×10^{-2}
Cluster 4 *vs.* all	PGS Cross psychiatric disorder	3.4	1.6×10^{-2}	8×10^{-2} (n.s.)
	PGS MDD	3.7	3×10^{-3}	1×10^{-2}
	PGS Schizophrenia	3.1	2×10^{-2}	1×10^{-1} (n.s.)
	PGS Educational attainment	-4.8	1×10^{-3}	5×10^{-3}
	PGS Neuroticism	3.5	6×10^{-3}	3×10^{-2}

Table S20. Replication – Significance testing with the WY procedure, *one-vs-one* comparisons

Only the comparisons with significant variables after the first adjustment are shown.
n.s.: not significant at an α=0.05 threshold. WY = Westfall and Young.

One-*vs*-One Comparison	Variable	t-statistic	*p*-value adjusted using Westfall and Young	*p*-value further adjusted for the number of comparisons (N=5)
Cluster 0 *vs.* Cluster 1	Age	-3.7	5×10^{-3}	5×10^{-2}
Cluster 0 *vs.* Cluster 4	PGS Cross psychiatric disorder	-4.5	1×10^{-3}	1×10^{-2}
	PGS MDD	-3.8	9×10^{-3}	9×10^{-2} (n.s.)
	PGS Schizophrenia	-4.4	1×10^{-3}	1×10^{-2}
	PGS Educational attainment	4.2	2×10^{-3}	2×10^{-2}
Cluster 1 *vs.* Cluster 2	Age	4.3	2×10^{-3}	2×10^{-2}
Cluster 1 *vs.* Cluster 4	PGS MDD	-3.4	1×10^{-2}	1×10^{-1} (n.s.)
	PGS Educational attainment	4.3	2×10^{-3}	2×10^{-2}
	PGS Neuroticism	-3.9	4×10^{-3}	4×10^{-2}
Cluster 2 *vs.* Cluster 3	Age	-3.3	2×10^{-2}	2×10^{-1} (n.s.)
Cluster 2 vs. Cluster 4	Age	-3.6	1×10^{-2}	1×10^{-1} (n.s.)
	PGS Educational attainment	3.6	1×10^{-2}	1×10^{-1} (n.s.)
Cluster 3 *vs.* Cluster 4	PGS Educational attainment	3.2	4×10^{-2}	4×10^{-1} (n.s.)

Table S21. PCA analysis with variables used for clustering

The table below shows the results of the PCA analysis performed on variables used for clustering with the purpose of exploring to which degree severity was captured by PC1.
The first component (PC1) explained 45.5% of the variance and correlated with the cluster labels (Spearman ρ=0.77). However, it predominantly captured the distinction between health and disease instead of the overall severity gradient: When removing cluster 0 and only correlating clusters 1-4 with PC1, the correlation dropped to ρ=0.37.

PC	Variance explained	Top five contributing variables				
1	45.5%	SCL90R global severity index	SCL90R depression	SCL90R Positive Symptom Total	STAIT	BDI Sum
2	5.8%	CTQ Emotional neglect	Maternal bonding	CTQ Physical neglect	CTQ Emotional abuse	CTQ Physical abuse
3	4.0%	VLMT Sum	Letter number span test	Corsi block-tapping test	Positive symptoms	NEOFFI Openness to experience
4	3.6%	NEOFFI Extraversion	Social support	NEOFFI Agreeableness	VLMT Sum	NEOFFI Openness to experience
5	2.9%	LEQ Positive Event Score	RSQ Fear of rejection or abandonment	SF36 Physical Functioning	IQ	YMRS
6	2.5%	YMRS	IQ	Positive symptoms	NEOFFI Openness to experience	NEOFFI Conscientiousness
7	2.4%	RSQ Preference for independence	RSQ Fear of Closeness	IQ	NEOFFI Conscientiousness	RSQ Fear of rejection or abandonment
8	2.2%	SF36 Bodily Pain	SF36 Physical Functioning	SF36 Role Physical	Negative symptoms	HAMD_Sum21
9	1.9%	LEQ Positive Event Score	NEOFFI Openness to experience	Corsi block-tapping test	Letter number span test	LEQ Negative Event Score
10	1.8%	RSQ Preference for independence	Corsi block-tapping test	CTQ Sexual abuse	Positive symptoms	LEQ Negative Event Score
11	1.5%	CTQ Sexual abuse	RSQ Fear of rejection or abandonment	YMRS	RSQ Preference for independence	NEOFFI Neuroticism
12	1.5%	YMRS	NEOFFI Openness to experience	Negative symptoms	LEQ Positive Event Score	Letter number span test
13	1.4%	CTQ Sexual abuse	IQ	Corsi block-tapping test	SAPS Composite Score	LEQ Negative Event Score
14	1.2%	NEOFFI Conscientiousness	RSQ Fear of rejection or abandonment	RSQ Desire for closeness	RSQ Preference for independence	SF36 Physical Functioning
15	1.1%	NEOFFI Agreeableness	HAMA Sum	NEOFFI Openness to experience	HAMD_Sum21	CTQ Sexual abuse
16	1.1%	NEOFFI Agreeableness	SF36 Role Physical	LEQ Negative Event Score	LEQ Positive Event Score	SF36 Social functioning

Table S22. Bipolar disorder subtypes across clusters.
For clusters 0-4, the percentages refer to the respective row-specific bipolar disorder (BD) subtype. NOS = not otherwise specified.

Variable	Full sample	Cluster 0	Cluster 1	Cluster 2	Cluster 3	Cluster 4
**Discovery sample**						
N (BD diagnosis)	41 (55%)	6 (15%)	0 (0%)	3 (7%)	18 (44%)	14 (34%)
Bipolar Type I, N (%)	33 (44%)	2 (6%)	1 (3%)	12 (36%)	8 (24%)	10 (30%)
Bipolar Type II, N (%)	1 (1%)	1 (100%)	0 (0%)	0 (0%)	0 (0%)	0 (0%)
Bipolar Disorder NOS, N (%)	41 (55%)	6 (15%)	0 (0%)	3 (7%)	18 (44%)	14 (34%)
**Replication sample**						
N (BD diagnosis)	44	1	9	7	17	10
Bipolar Type I, N (%)	21 (48%)	0 (0%)	5 (24%)	2 (10%)	8 (38%)	6 (29%)
Bipolar Type II, N (%)	23 (52%)	1 (4%)	4 (17%)	5 (22%)	9 (39%)	4 (17%)
Bipolar Disorder NOS, N (%)	0 (0%)	0 (0%)	0 (0%)	0 (0%)	0 (0%)	0 (0%)

Table S23. Assessing psychotic symptoms of MDD and BD patients in cluster 4

Comparison of positive symptoms scores [mean score (SD)] of MDD and BD patients in cluster 4 to MDD/BD patients in the other clusters. Statistical significance was assessed using one-sided t-tests with the hypothesis that cluster 4 patients show more positive symptoms. Abbreviation: n.s. = *p*>0.05.

	**MDD**			**BD**		
**Variable**	Cluster 4	Clusters 0-3	*t-test (one-sided)*	Cluster 4	Clusters 0-3	*t-test (one-sided)*
N	105	372		24	51	
Positive Symptoms	2.0 (4.4)	0.4 (1.3)	t = 3.6, p = 0.0003	5.4 (7.2)	1.4 (2.4)	t = 2.6, p = 0.007
Delusions	0.5 (1.9)	0.09 (0.5)	t = 2.4 p = 0.01	0.7 (1.6)	0.1 (0.5)	t = 1.7 p = 0.05
Hallucinations	0.3 (1.4)	0.03 (0.2)	t = 1.8 p = 0.04	0.1 (0.3)	0.02 (0.1)	t = 1.0, p = 0.1 (n.s.)
Bizarre Behavior	0.2 (0.9)	0.06 (0.4)	t = 1.5, p = 0.06 (n.s.)	0.5 (1.2)	0.04 (0.3)	t = 1.9, p = 0.03
Positive Formal Thought Disorder	1.0 (2.7)	0.3 (1.0)	t = 2.7 p = 0.004	4.1 (5.6)	1.3 (2.2)	t = 2.4 p = 0.01

Table S24. Healthy Controls – clinical assessment

This table shows phenotypic characteristics of Healthy controls, constituting a secondary, descriptive analysis to assess the heterogeneity within healthy controls.
The significance of genetic variables was tested using the Westfall and Young procedure (Methods S6). Here, only the significant variables are shown.
Importantly, the number of healthy controls differed strongly between clusters, leading to a large class imbalance and resulting in a lack of power for the analysis of healthy controls on their own.
Abbreviations: n.s. = adjusted *p*>0.05.

Please see the separate Excel file for the table.

Figure S1. Correlation plot of variables used for clustering

The figure below shows the (Spearman) correlation between the 57 variables used for clustering. Even though these features are correlated, the HDDC algorithm does not require prior dimensionality reduction. It can handle higher dimensions and correlated features [19].

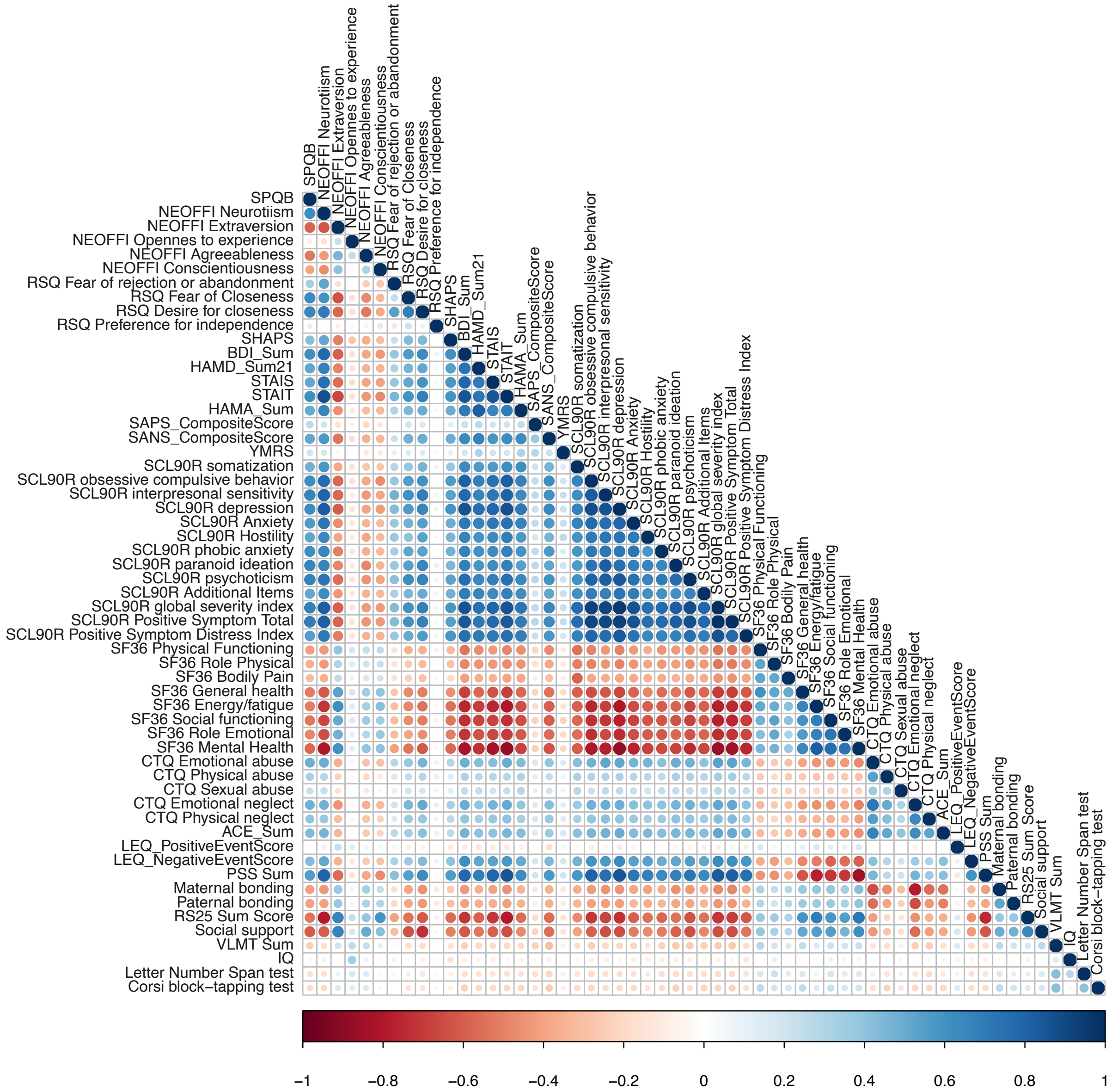

Figure S2. Population substructure (genetic ancestry components).

Post-QC, pre-imputation genotype data were used to calculate ancestry components (for details on the QC, see the Methods S2).
Additional variant filtering steps were: Removal of variants with a MAF <0.05 or HWE *p*-value $<10^{-3}$; removal of variants mapping to the extended major histocompatibility region (chromosome 6, 25-35 Mbp) or to a typical inversion site on chromosome 8 (7-13 Mbp); linkage disequilibrium (LD) pruning (command `--indep-pairwise 200 100 0.2`).
Next, the pairwise identity-by-state (IBS) matrix of all individuals was calculated using the command `--genome` on the filtered genotype data. Multidimensional scaling (MDS) analysis was performed on the IBS matrix using the eigendecomposition-based algorithm in PLINK v1.90.
Abbreviations: HC, healthy control; MDD, major depressive disorder; BD, bipolar disorder; SZA, schizoaffective disorder; SCZ, schizophrenia; OTH, other psychiatric diagnoses.

A: Population substructure in the discovery sample.

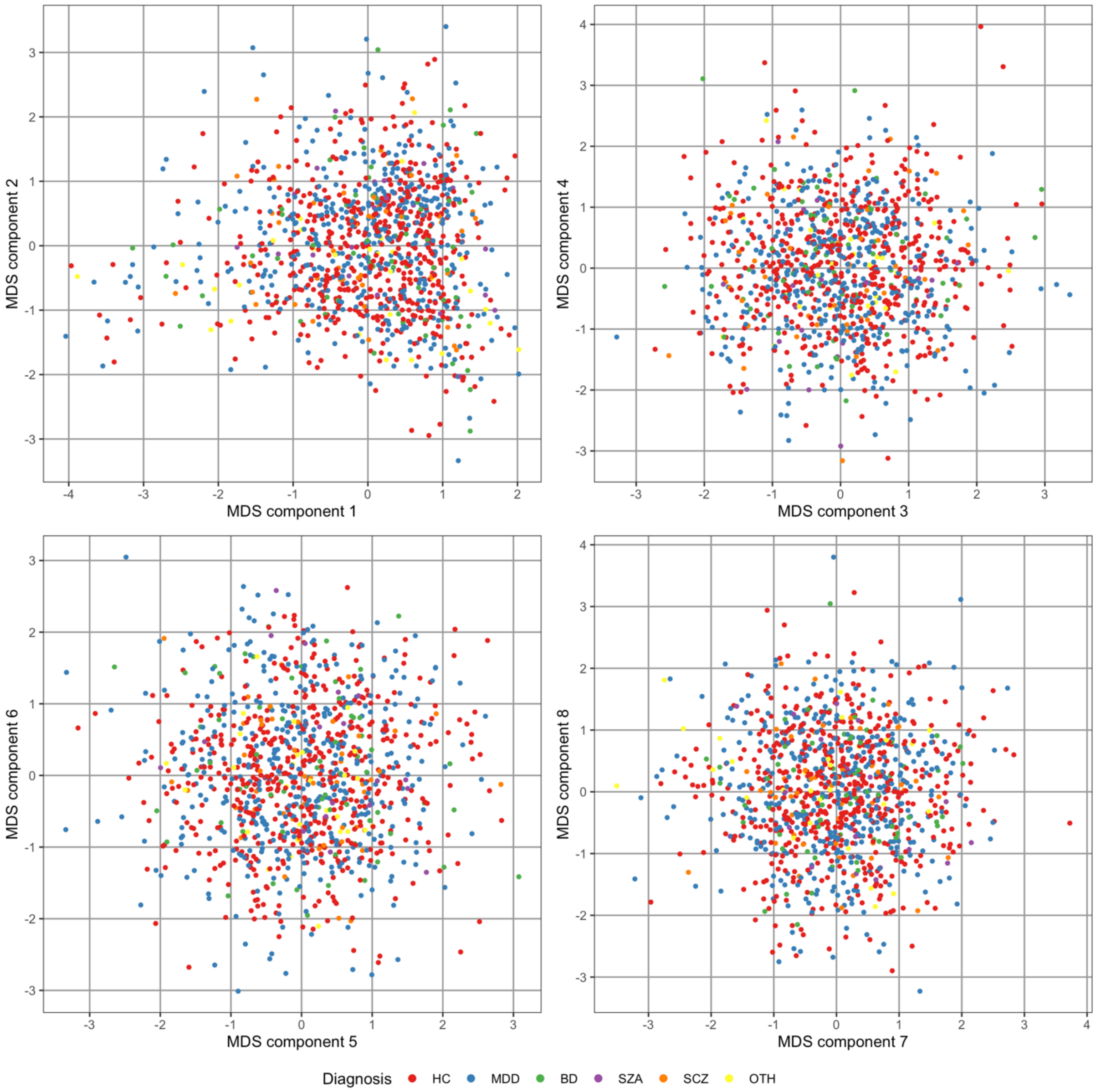

B: Population substructure in the replication sample.

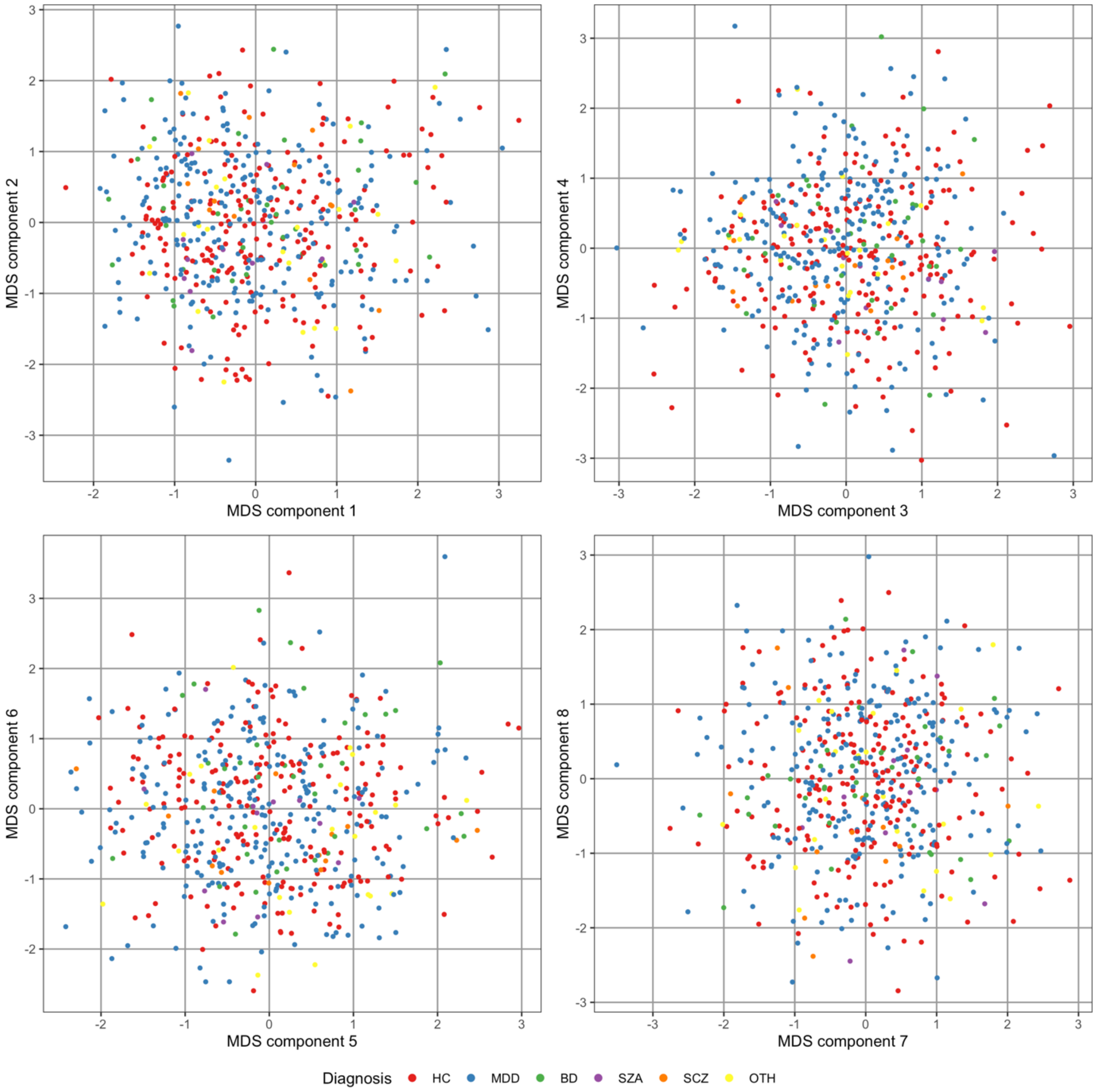

Figure S3. Overview of the clustering pipeline

Abbreviations: ICL, Integrated Likelihood criterion [21].
The figure accompanies the clustering procedure described in detail in the Supplementary Methods S4. The results of each step are shown in Figure S4.
Note that steps 1-4 used different resampling strategies to reduce the risk of overfitting, which is higher when employing only one approach. K=5 was selected as the optimal choice in all steps.

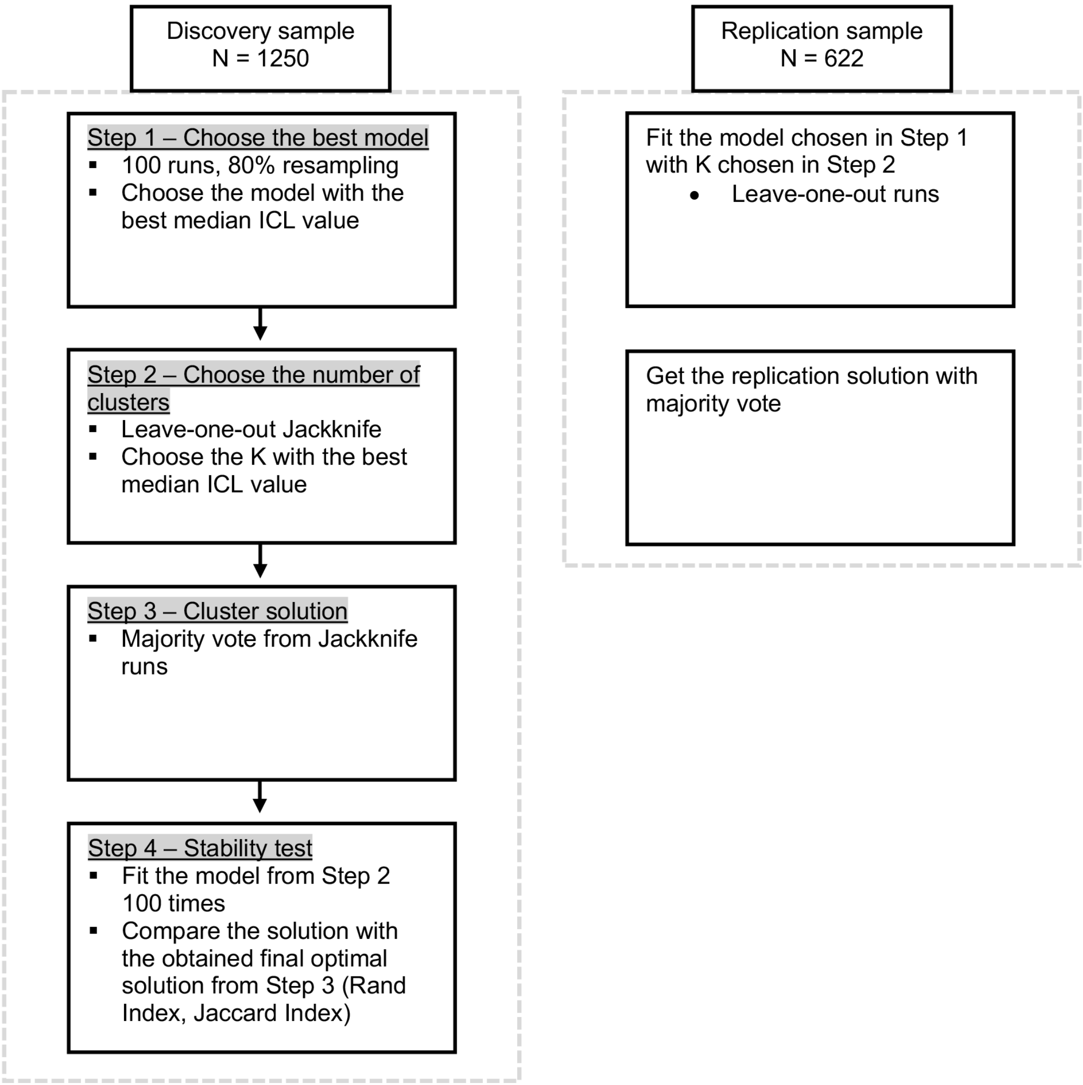

Figure S4. Results from the clustering pipeline

The plot shows results of each step of the clustering procedure, as described in detail in Methods S4 (the corresponding workflow is shown in Figure S3).
Note that steps A-C used different resampling strategies to reduce the risk of overfitting, which is higher when employing only one approach. K=5 was selected as the optimal choice in all three steps.
A: Step 1: Choosing the best fitting model. Two groups of models were created (separated by a substantial difference in median ICL). The group achieving better fits (ICL values) consisted of models allowing for modeling of class-specific noise. Among these models, the model AkBkQkDk achieved the highest median ICL value and was submitted to the second step of the pipeline (see Figure S3).
The model AkBkQkDk has the following characteristics:
Ak: The classes have different parameters but there is only one parameter per class
Bk: Each class has its proper noise
Qk: All classes have their proper orientation matrix
Dk: The dimensions are free and proper to each class
In the context of the data, the optimal model that was chosen assumes that there is a cluster-specific variance (Ak), cluster-specific noise (Bk), and that clusters can have different orientations (Qk). Since the HDDC algorithm is the subspace clustering algorithm based on the assumption that the clusters live in the subspaces of a lower dimension than the initial one, Dk is in this context considered to be the dimension of the cluster-specific subspace. For more theoretical details, see Bouveyron *et al.* [19].
B: Step 2: Choosing the optimal number of clusters K. Error bars show median absolute deviations.
C: Stability test: K = 5 confirmed as an optimal solution. Error bars show median absolute deviations.
D: Stability measures – Rand and Jaccard indices. Stability labels were obtained with the majority voting scheme from the stability analysis runs and compared to the final cluster labels.

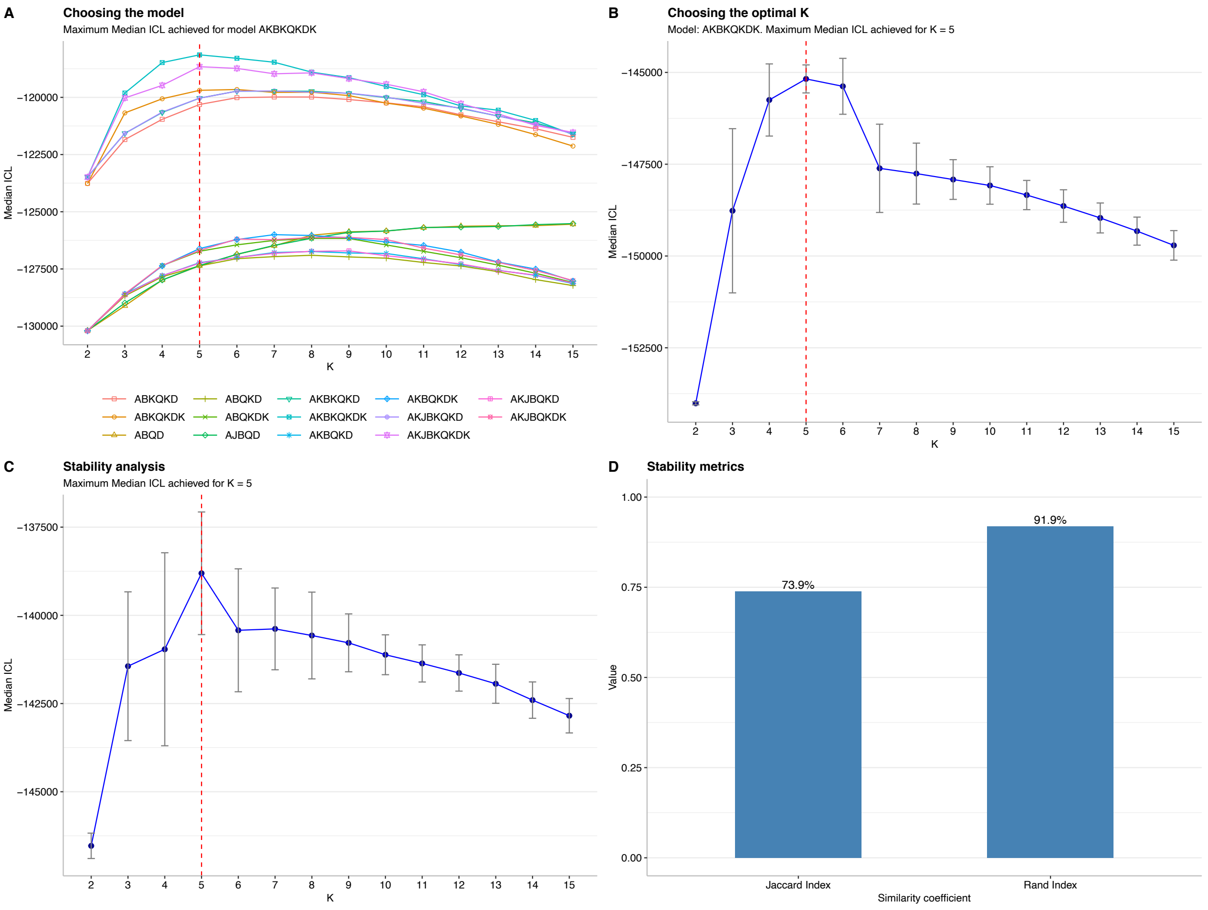

Figure S5. Distributions of the most important variables per cluster

The figures below show the most important variables for each cluster, based on the HDDA analysis shown in Table S5. A horizontal line represents the mean, and the error bars indicate the standard deviation. The dot size is proportional to the number of individuals with the given value.
A: Distributions of the two most important variables per cluster. The dark square on top indicates the cluster for which the variable was among the top two most important variables (see Table S5).
B: Distributions of all other variables from Table S5, in alphabetical order.

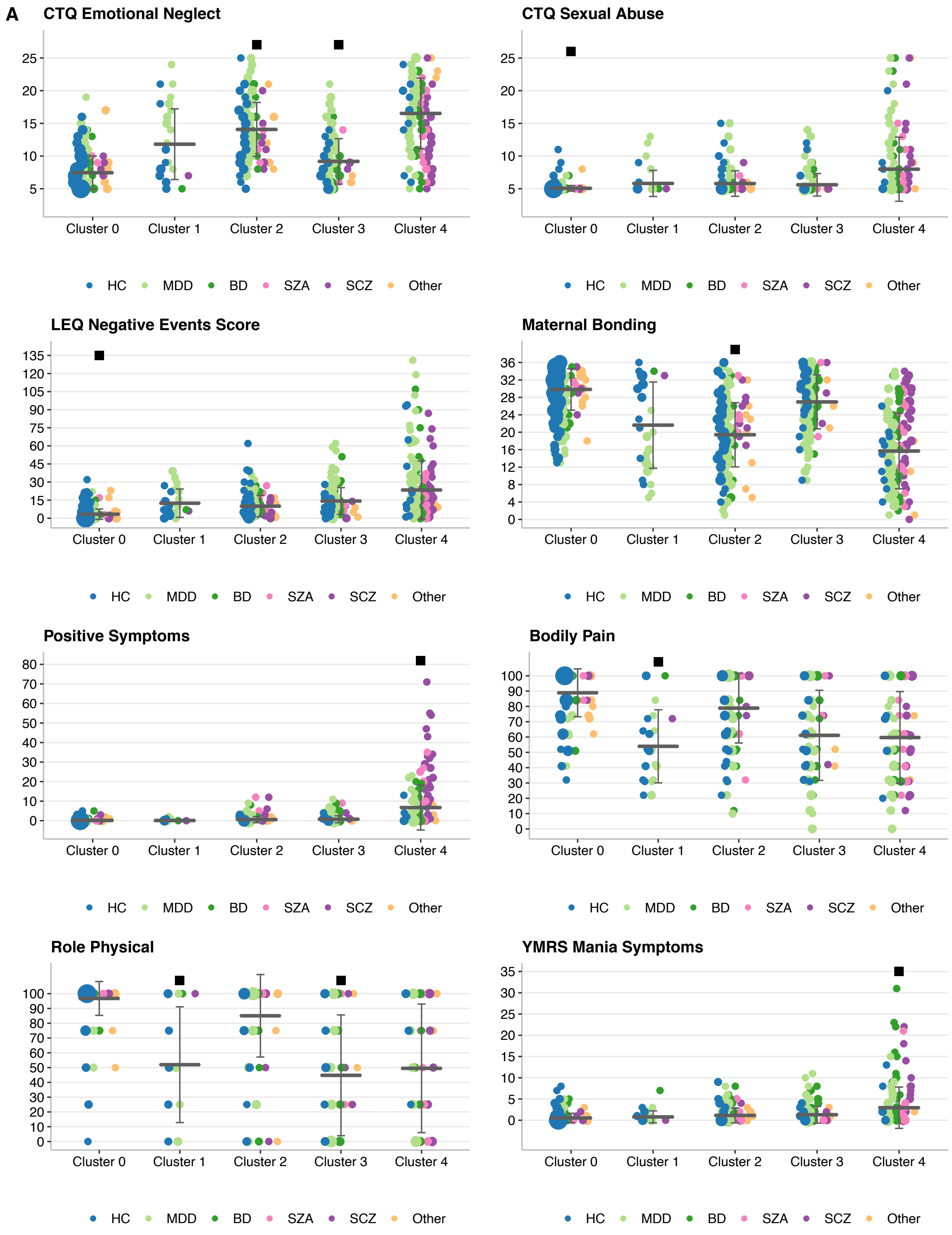

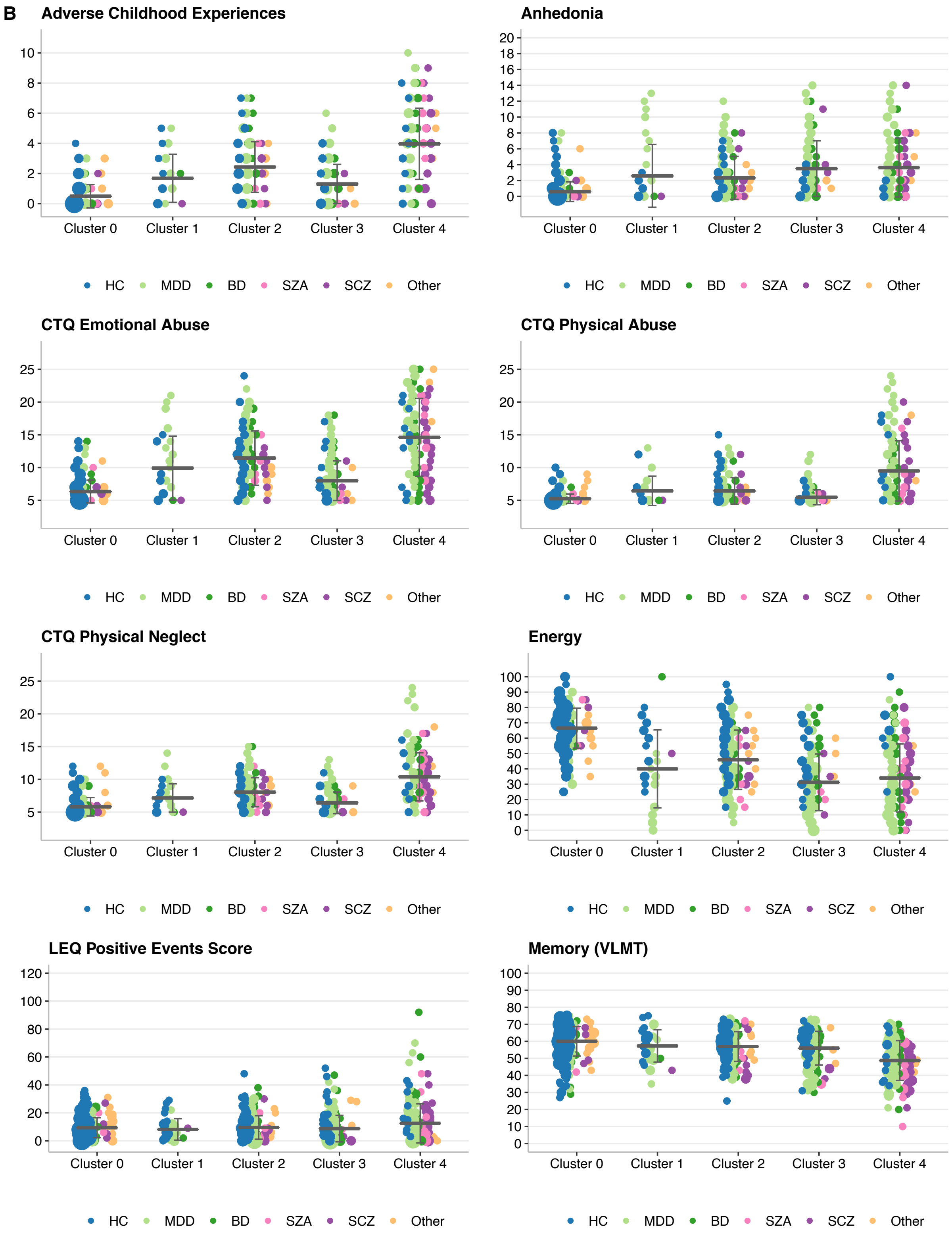
B
Adverse Childhood Experiences
Anhedonia
CTQ Emotional Abuse
CTQ Physical Abuse
CTQ Physical Neglect
Energy
LEQ Positive Events Score
Memory (VLMT)
Cluster 0
Cluster 1
Cluster 2
Cluster 3
Cluster 4
HC
MDD
BD
SZA
SCZ
Other

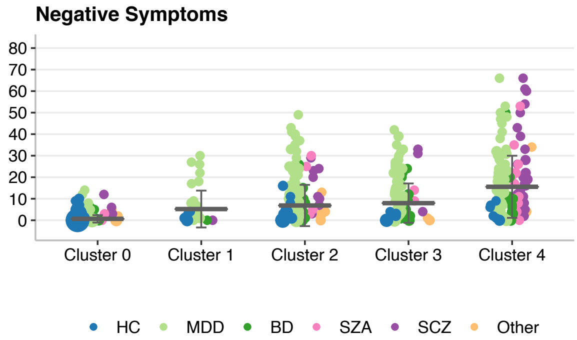
Negative Symptoms
80
70
60
50
40
30
20
10
0
Cluster 0
Cluster 1
Cluster 2
Cluster 3
Cluster 4
HC
MDD
BD
SZA
SCZ
Other

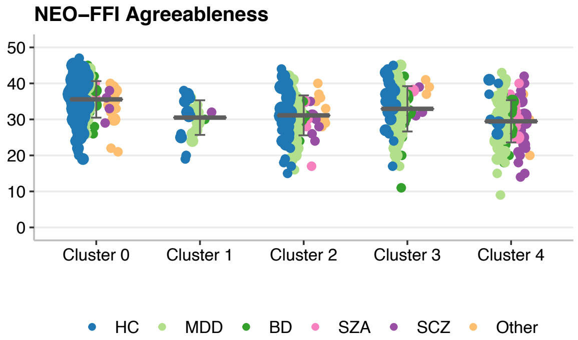
NEO−FFI Agreeableness
50
40
30
20
10
0
Cluster 0
Cluster 1
Cluster 2
Cluster 3
Cluster 4
HC
MDD
BD
SZA
SCZ
Other

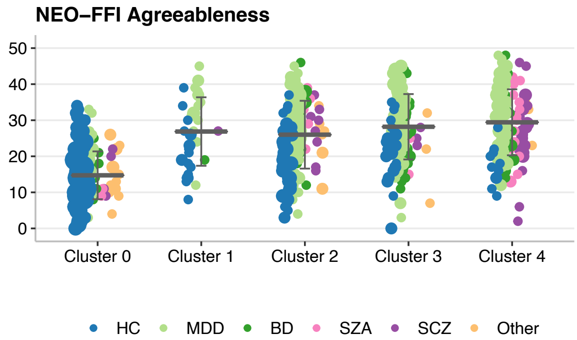
NEO−FFI Agreeableness
50
40
30
20
10
0
Cluster 0
Cluster 1
Cluster 2
Cluster 3
Cluster 4
HC
MDD
BD
SZA
SCZ
Other

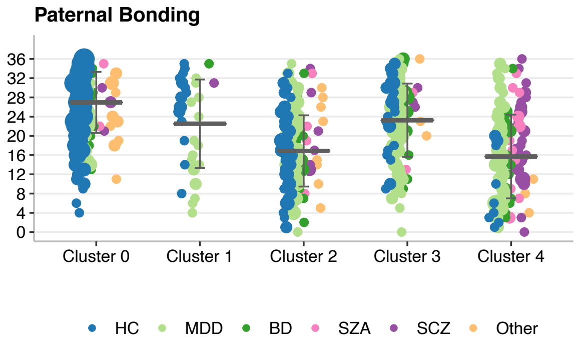
Paternal Bonding
36
32
28
24
20
16
12
8
4
0
Cluster 0
Cluster 1
Cluster 2
Cluster 3
Cluster 4
HC
MDD
BD
SZA
SCZ
Other

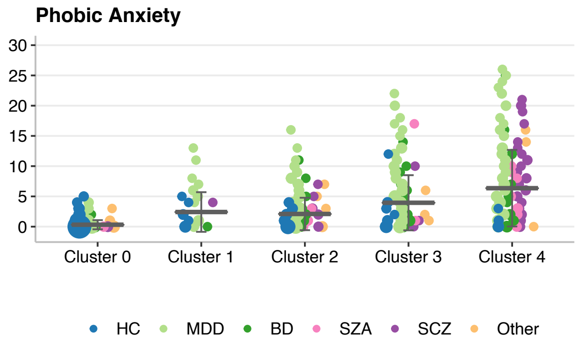
Phobic Anxiety
30
25
20
15
10
5
0
Cluster 0
Cluster 1
Cluster 2
Cluster 3
Cluster 4
HC
MDD
BD
SZA
SCZ
Other

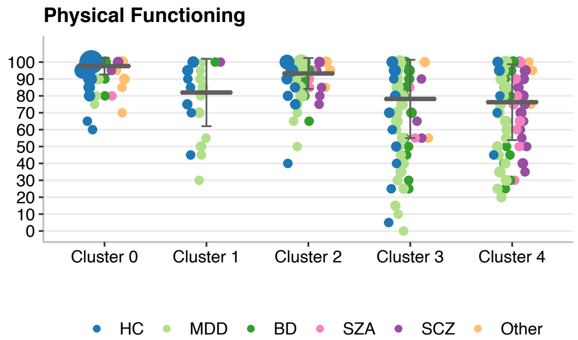
Physical Functioning
100
90
80
70
60
50
40
30
20
10
0
Cluster 0
Cluster 1
Cluster 2
Cluster 3
Cluster 4
HC
MDD
BD
SZA
SCZ
Other

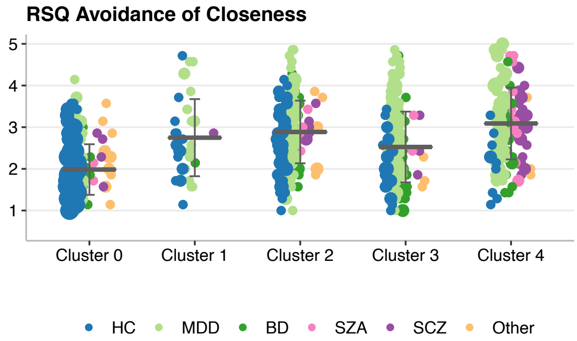
RSQ Avoidance of Closeness
5
4
3
2
1
Cluster 0
Cluster 1
Cluster 2
Cluster 3
Cluster 4
HC
MDD
BD
SZA
SCZ
Other

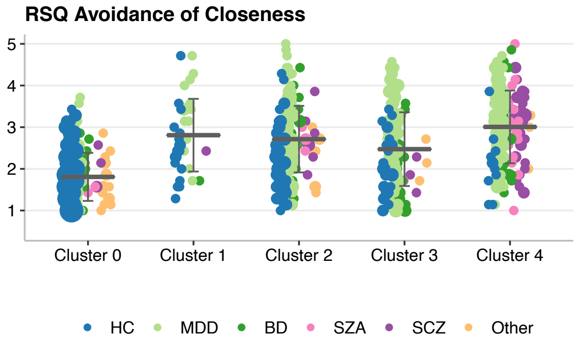
RSQ Avoidance of Closeness
5
4
3
2
1
Cluster 0
Cluster 1
Cluster 2
Cluster 3
Cluster 4
HC
MDD
BD
SZA
SCZ
Other

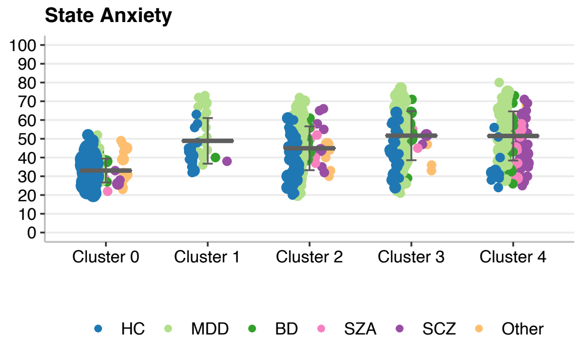
State Anxiety
100
90
80
70
60
50
40
30
20
10
0
Cluster 0
Cluster 1
Cluster 2
Cluster 3
Cluster 4
HC
MDD
BD
SZA
SCZ
Other

Figure S6. Distribution of PGS for MDD patients

The plot shows only PGS that were significant after Bonferroni correction (adjusted *p*<0.05) in *one-vs-all* analyses using the Westfall and Young procedure (Methods S6) **in the discovery-stage analysis of MDD patients** (Table S8). PGS were standardized by Z-score transformation, the y-axis unit is standard deviations. A horizontal line represents the mean and the error bars indicate the standard deviation of the variables in the respective clusters.
Panels A-B show results from the discovery and C-D from the replication sample.

A: Attention-deficit/hyperactivity disorder PGS in discovery-stage MDD patients**,** significantly higher for the MDD patients in cluster 4 compared to the MDD patients in all other clusters (adjusted *p*=0.0095). The ADHD PGS was not significant in the analysis including all diagnoses**. B:** Educational attainment PGS in discovery-stage MDD patients, significantly lower for the MDD patients in cluster 4 compared to the MDD patients in all other clusters (adjusted *p*=0.005)**. C**: ADHD PGS for MDD patients in the replication sample, The discovery-stage association (significantly higher ADHD PGS in cluster 4 compared to all other clusters) did not replicate. **D:** PGS Educational attainment for MDD patients in the replication sample. The significant difference of MDD patients in cluster 4 against all other MDD patients replicated (Bonferroni-adjusted *p*=0.005).

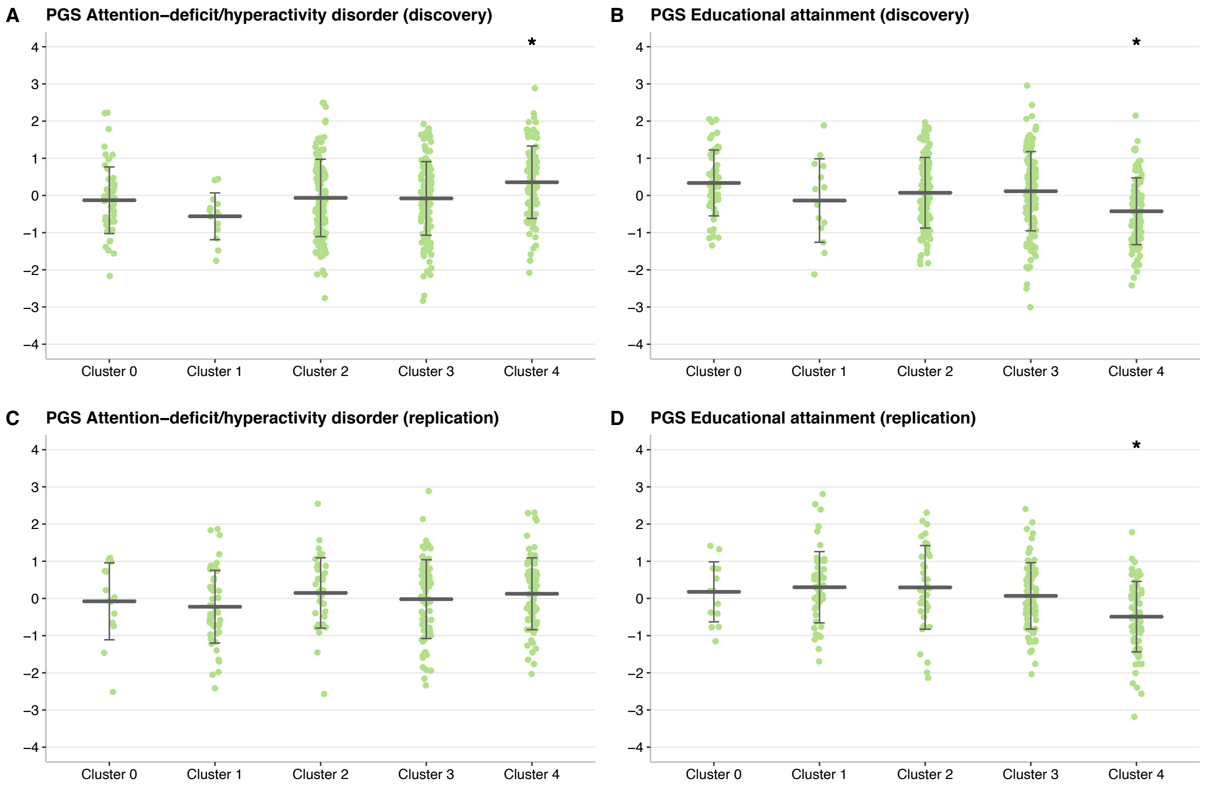

Figure S7. Discovery-stage HDDA models projected to the replication sample

The projection of HDDA models was conducted to match the discovery and replication clusters, as explained in the Supplementary Methods S8. The matched discovery-stage and replication clusters achieving the prediction>70% are represented with colored bars. All models except *Cluster 1-vs-all* showed higher predictions to the replication sample for one of the projections.
The balanced accuracy was used as the evaluation metric for the prediction. This metric evaluates a binary classifier by accounting for the imbalance in classes. It is calculated as the average of the proportion of correctly classified individuals from each class: (true positive rate + true negative rate)/2.

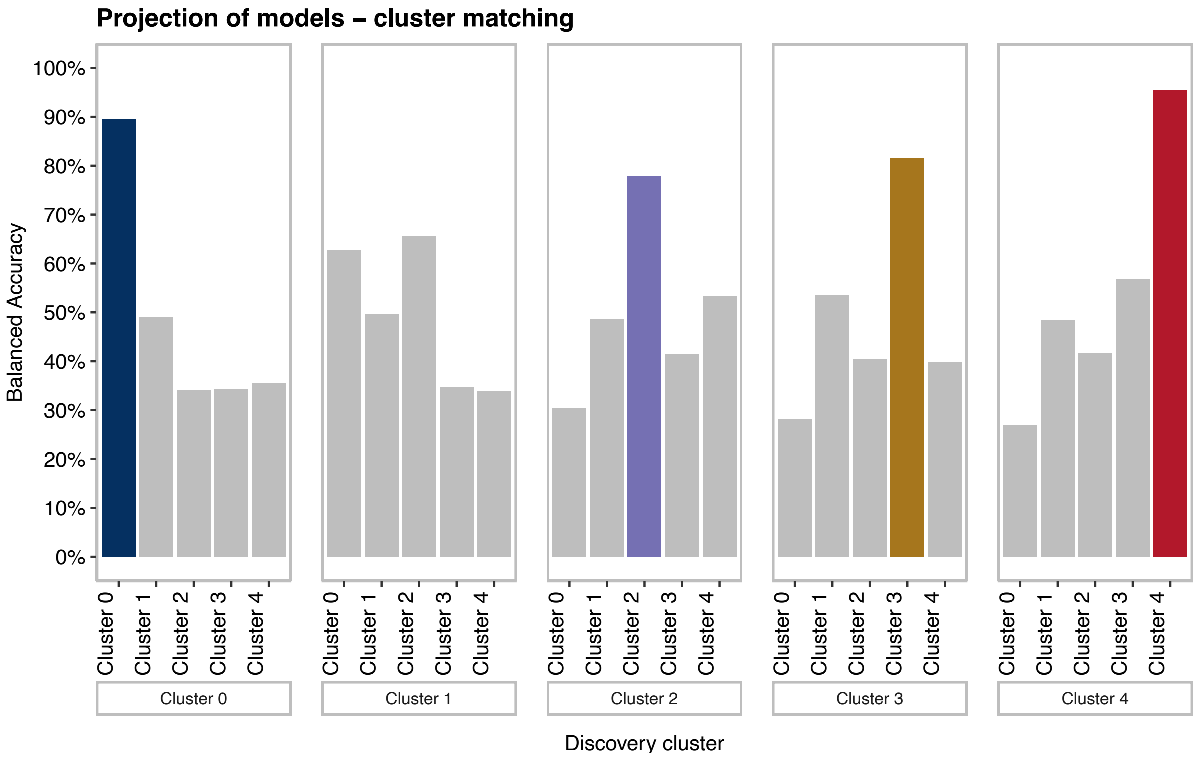

Figure S8. Distribution of ADHD PGS for healthy controls

The plot shows the ADHD PGS distribution for **healthy controls only**. The ADHD PGS was the PGS showing the significant difference after the W-Y adjustment (Table S24). However, it does not pass the Bonferroni correction. One needs to be aware of the big class imbalance when observing healthy controls across different clusters and, thus, possible lack of power.

PGS were standardized by Z-score transformation, the y-axis unit is standard deviations. A horizontal line represents the mean and the error bars indicate the standard deviation of the variables in the respective clusters.

A: Attention-deficit/hyperactivity disorder PGS in discovery-stage healthy controls patients**,** significantly different after W-Y adjustment for cluster 0 vs. all (*p*=0.02) and cluster 2 vs. all. (*p*=0.05)
B: Attention-deficit/hyperactivity disorder PGS in replication-stage healthy controls patients

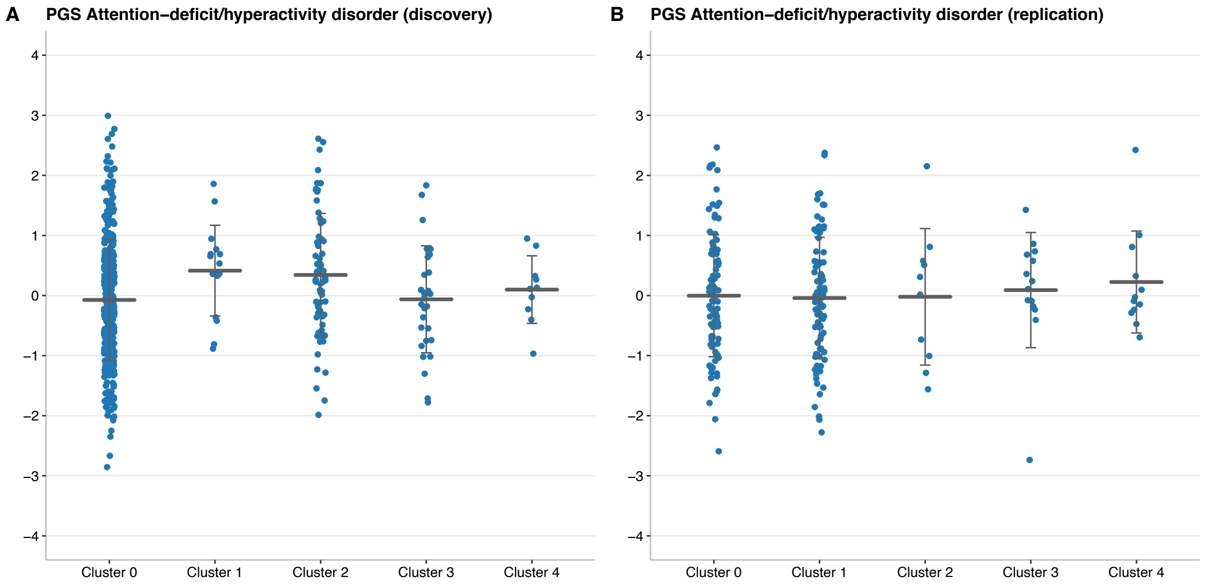

Full FOR2107 acknowledgements

This work is part of the German multicenter consortium “Neurobiology of Affective Disorders. A translational perspective on brain structure and function“, funded by the German Research Foundation (Deutsche Forschungsgemeinschaft DFG; Forschungsgruppe/Research Unit FOR2107).
Principal investigators (PIs) with respective areas of responsibility in the FOR2107 consortium are: Work Package WP1, FOR2107/MACS cohort and brainimaging: Tilo Kircher (speaker FOR2107; DFG grant numbers KI 588/14-1, KI 588/14-2), Udo Dannlowski (co-speaker FOR2107; DA 1151/5-1, DA 1151/5-2), Axel Krug (KR 3822/5-1, KR 3822/7-2), Igor Nenadic (NE 2254/1-2), Carsten Konrad (KO 4291/3-1). WP2, animal phenotyping: Markus Wöhr (WO 1732/4-1, WO 1732/4-2), Rainer Schwarting (SCHW 559/14-1, SCHW 559/14-2). WP3, miRNA: Gerhard Schratt (SCHR 1136/3-1, 1136/3-2). WP4, immunology, mitochondriae: Judith Alferink (AL 1145/5-2), Carsten Culmsee (CU 43/9-1, CU 43/9-2), Holger Garn (GA 545/5-1, GA 545/7-2). WP5, genetics: Marcella Rietschel (RI 908/11-1, RI 908/11-2), Markus Nöthen (NO 246/10-1, NO 246/10-2), Stephanie Witt (WI 3439/3-1, WI 3439/3-2). WP6, multimethod data analytics: Andreas Jansen (JA 1890/7-1, JA 1890/7-2), Tim Hahn (HA 7070/2-2), Bertram Müller-Myhsok (MU1315/8-2), Astrid Dempfle (DE 1614/3-1, DE 1614/3-2). CP1, biobank: Petra Pfefferle (PF 784/1-1, PF 784/1-2), Harald Renz (RE 737/20-1, 737/20-2). CP2, administration. Tilo Kircher (KI 588/15-1, KI 588/17-1), Udo Dannlowski (DA 1151/6-1), Carsten Konrad (KO 4291/4-1).
Data access and responsibility: All PIs take responsibility for the integrity of the respective study data and their components. All authors and coauthors had full access to all study data.
Acknowledgements and members by Work Package (WP): WP1: Henrike Bröhl, Katharina Brosch, Bruno Dietsche, Rozbeh Elahi, Jennifer Engelen, Sabine Fischer, Jessica Heinen, Svenja Klingel, Felicitas Meier, Tina Meller, Julia-Katharina Pfarr, Kai Ringwald, Torsten Sauder, Simon Schmitt, Frederike Stein, Annette Tittmar, Dilara Yüksel (Dept. of Psychiatry, Marburg University). Mechthild Wallnig, Rita Werner (Core-Facility Brainimaging, Marburg University). Carmen Schade-Brittinger, Maik Hahmann (Coordinating Centre for Clinical Trials, Marburg). Michael Putzke (Psychiatric Hospital, Friedberg). Rolf Speier, Lutz Lenhard (Psychiatric Hospital, Haina). Birgit Köhnlein (Psychiatric Practice, Marburg). Peter Wulf, Jürgen Kleebach, Achim Becker (Psychiatric Hospital Hephata, Schwalmstadt-Treysa). Ruth Bär (Care facility Bischoff, Neukirchen). Matthias Müller, Michael Franz, Siegfried Scharmann, Anja Haag, Kristina Spenner, Ulrich Ohlenschläger (Psychiatric Hospital Vitos, Marburg). Matthias Müller, Michael Franz, Bernd Kundermann (Psychiatric Hospital Vitos, Gießen). Christian Bürger, Katharina Dohm, Fanni Dzvonyar, Verena Enneking, Stella Fingas, Katharina Förster, Janik Goltermann, Dominik Grotegerd, Hannah Lemke, Susanne Meinert, Nils Opel, Ronny Redlich, Jonathan Repple, Kordula Vorspohl, Bettina Walden, Dario Zaremba (Dept. of Psychiatry, University of Münster). Harald Kugel, Jochen Bauer, Walter Heindel, Birgit Vahrenkamp (Dept. of Clinical Radiology, University of Münster). Gereon Heuft, Gudrun Schneider (Dept. of Psychosomatics and Psychotherapy, University of Münster). Thomas Reker (LWL-Hospital Münster). Gisela Bartling (IPP Münster). Ulrike Buhlmann (Dept. of Clinical Psychology, University of Münster). WP5: Helene Dukal, Christine Hohmeyer, Lennard Stütz, Viola Lahr, Fabian Streit, Josef Frank, Lea Sirignano (Dept. of Genetic Epidemiology, Central Institute of Mental Health, Medical Faculty Mannheim, Heidelberg University). Stefanie Heilmann-Heimbach, Stefan Herms, Per Hoffmann (Institute of Human Genetics, University of Bonn, School of Medicine & University Hospital Bonn). Andreas J. Forstner (Institute of Human Genetics, University of Bonn, School of Medicine & University Hospital Bonn; Centre for Human Genetics, Marburg University). WP6: Anastasia Benedyk, Miriam Bopp, Roman Keßler, Maximilian Lückel, Verena Schuster, Christoph Vogelbacher (Dept. of Psychiatry, Marburg University). Jens Sommer, Olaf Steinsträter (Core-Facility Brainimaging, Marburg University). Thomas W.D. Möbius (Institute of Medical Informatics and Statistics, Kiel University). CP1: Julian Glandorf, Fabian Kormann, Arif Alkan, Fatana Wedi, Lea Henning, Alena Renker, Karina Schneider, Elisabeth Folwarczny, Dana Stenzel, Kai Wenk, Felix Picard, Alexandra Fischer, Sandra Blumenau, Beate Kleb, Doris Finholdt, Elisabeth Kinder, Tamara Wüst, Elvira Przypadlo, Corinna Brehm (Comprehensive Biomaterial Bank Marburg, Marburg University).
The FOR2107 cohort project (WP1) was approved by the Ethics Committees of the Medical Faculties, University of Marburg (AZ: 07/14) and University of Münster (AZ: 2014-422-b-S). The study was supported by the German Federal Ministry of Education and Research (BMBF), through the Integrated Network IntegraMent, under the auspices of the e:Med programme (grants 01ZX1314A/01ZX1614A to MMN; 01ZX1314G/01ZX1614G to MR; 01ZX1614J to BMM) through grants 01EE1406C to MR and 01EE1409C to MR and SHW, and through ERA-NET NEURON, “SynSchiz - Linking synaptic dysfunction to disease mechanisms in schizophrenia - a multilevel investigation“ (01EW1810 to MR) and BMBF grants 01EE1409C and 01EE1406C to MR and SHW.

Supplementary Materials References

1. Meller T, Schmitt S, Stein F, Brosch K, Mosebach J, Yüksel D, et al. Associations of schizophrenia risk genes ZNF804A and CACNA1C with schizotypy and modulation of attention in healthy subjects. Schizophr Res. 2019. 7 May 2019.
2. Chang CC, Chow CC, Tellier LCAM, Vattikuti S, Purcell SM, Lee JJ. Second-generation PLINK: rising to the challenge of larger and richer datasets. Gigascience. 2015;4.
3. Andlauer TFM, Buck D, Antony G, Bayas A, Bechmann L, Berthele A, et al. Novel multiple sclerosis susceptibility loci implicated in epigenetic regulation. Sci Adv. 2016;2:e1501678–e1501678.
4. Delaneau O, Zagury J-F, Marchini J. Improved whole-chromosome phasing for disease and population genetic studies. Nat Methods. 2013;10:5–6.
5. Howie B, Fuchsberger C, Stephens M, Marchini J, Abecasis GR. Fast and accurate genotype imputation in genome-wide association studies through pre-phasing. Nat Genet. 2012;44:955–959.
6. Howie BN, Donnelly P, Marchini J. A flexible and accurate genotype imputation method for the next generation of genome-wide association studies. PLoS Genet. 2009;5:e1000529–e1000529.
7. Demontis D, Walters RK, Martin J, Mattheisen M, Als TD, Agerbo E, et al. Discovery of the first genome-wide significant risk loci for attention deficit/hyperactivity disorder. Nat Genet. 2019;51:63–75.
8. Grove J, Ripke S, Als TD, Mattheisen M, Walters RK, Won H, et al. Identification of common genetic risk variants for autism spectrum disorder. Nat Genet. 2019;51:431–444.
9. Stahl EA, Breen G, Forstner AJ, McQuillin A, Ripke S, Trubetskoy V, et al. Genome-wide association study identifies 30 loci associated with bipolar disorder. Nat Genet. 2019;51:793–803.
10. Lee P, Anttila V, Won H, Feng Y-C, Rosenthal J, Zhu Z, et al. Genomic Relationships, Novel Loci, and Pleiotropic Mechanisms across Eight Psychiatric Disorders. Cell. 2019;179:1469–1482.
11. Okbay A, Beauchamp JP, Fontana MA, Lee JJ, Pers TH, Rietveld CA, et al. Genome-wide association study identifies 74 loci associated with educational attainment. Nature. 2016;533:539–542.
12. van den Berg SM, de Moor MHM, Verweij KJH, Krueger RF, Luciano M, Arias Vasquez A, et al. Meta-analysis of Genome-Wide Association Studies for Extraversion: Findings from the Genetics of Personality Consortium. Behav Genet. 2016;46:170–182.
13. Baselmans BML, Bartels M. A genetic perspective on the relationship between eudaimonic - and hedonic well-being. Sci Rep. 2018;8:14610.
14. Howard DM, Adams MJ, Clarke T-K, Hafferty JD, Gibson J, Shirali M, et al. Genome-wide meta-analysis of depression identifies 102 independent variants and highlights the importance of the prefrontal brain regions. Nat Neurosci. 2019;22:343–352.
15. Luciano M, Hagenaars SP, Davies G, Hill WD, Clarke T-K, Shirali M, et al. Association analysis in over 329,000 individuals identifies 116 independent variants influencing neuroticism. Nat Genet. 2018;50:6–11.
16. Pardiñas AF, Holmans P, Pocklington AJ, Escott-Price V, Ripke S, Carrera N, et al. Common schizophrenia alleles are enriched in mutation-intolerant genes and in regions under strong background selection. Nat Genet. 2018;50:381–389.
17. Ge T, Chen C-Y, Ni Y, Feng Y-CA, Smoller JW. Polygenic prediction via Bayesian regression and continuous shrinkage priors. Nat Commun. 2019;10:1776.
18. Andlauer TFM, Guzman-Parra J, Streit F, Strohmaier J, González MJ, Gil Flores S, et al. Bipolar multiplex families have an increased burden of common risk variants for psychiatric disorders. Mol Psychiatry. 2019. 2019. https://doi.org/10.1038/s41380-019-0558-2.
19. Bouveyron C, Girard S, Schmid C. High-dimensional data clustering. Comput Stat Data Anal. 2007;52:502–519.
20. Berg L, Bouveyron C, Girard S. HDclassif: An R Package for Model-Based Clustering and Discriminant Analysis of High-Dimensional Data. J Stat Softw. 2012;46.
21. Bertoletti M, Friel N, Rastelli R. Choosing the number of clusters in a finite mixture model using an exact Integrated Completed Likelihood criterion:1–23.
22. Hawkins DM, Basak SC, Mills D. Assessing model fit by cross-validation. J Chem Inf Comput Sci. 2003;43:579–586.
23. Chiu D, Talhouk A. DiceR: An R package for class discovery using an ensemble driven approach. BMC Bioinformatics. 2018;19.
24. Bouveyron C, Girard S, Schmid C. High-Dimensional Discriminant Analysis. Commun Stat -

Theory Methods. 2007;36:2607–2623.
25. Robin X, Turck N, Hainard A, Tiberti N, Lisacek F, Sanchez J-C, et al. pROC: an open-source package for R and S+ to analyze and compare ROC curves. BMC Bioinformatics. 2011;12:77.
26. Tibshirani R. Regression Shrinkage and Selection via the Lasso. J R Stat Soc (Series B). 1996;58:267–288.
27. Friedman J, Hastie T, Tibshirani R. Regularization Paths for Generalized Linear Models via Coordinate Descent. J Stat Software, Artic. 2010;33.
28. López-Ratón M, Rodríguez-Álvarez MX, Cadarso-Suárez C, Gude-Sampedro F. OptimalCutpoints: An R Package for Selecting Optimal Cutpoints in Diagnostic Tests. J Stat Software; Vol 1, Issue 8. 2014. 13 November 2014.
29. Pollard KS, Dudoit S, van der Laan MJ. Multiple Testing Procedures: R multtest Package and Applications to Genomics, in Bioinformatics and Computational Biology Solutions Using R and Bioconductor. Springer; 2005.
30. Yee TW. The VGAM Package for Categorical Data Analysis. J Stat Software, Artic. 2010;32.
31. Huang H, Liu Y, Yuan M, Marron JS. Statistical significance of clustering using soft thresholding. J Comput Graph Stat. 2015;24:975–993.
